# FIRST-ICU: forecasting interventions and risk stratification in the ICU using graph neural network autoencoders

**DOI:** 10.1038/s41746-026-02890-1

**Published:** 2026-06-11

**Authors:** Nosa Aikodon, Ivan Olier, Brian W. Johnston, Ingeborg D. Welters, Gregory Y. H. Lip, Sandra Ortega-Martorell

**Affiliations:** 1 Artificial Intelligence and Digital Technologies Research Institute, Liverpool John Moores University, Liverpool, UK; 2 Liverpool Centre for Cardiovascular Science at University of Liverpool, Liverpool John Moores University and Liverpool Heart & Chest Hospital, Liverpool, UK; 3 Liverpool University Hospitals NHS Foundation Trust, Liverpool, UK; 4 Department of Cardiovascular and Metabolic Medicine, Institute of Life Course and Medical Sciences, University of Liverpool, Liverpool, UK; 5 Department of Clinical Medicine, Aalborg University, Aalborg, Denmark; 6 Medical University of Bialystok, Bialystok, Poland

**Keywords:** Computational biology and bioinformatics, Diseases, Health care, Medical research

## Abstract

Critically ill patients frequently require multiple concurrent interventions with complex interdependencies, yet existing prediction models treat these as independent events. We developed FIRST-ICU (Forecasting Interventions and Risk Stratification in the ICU), a unified deep learning framework integrating a graph neural network encoder, LSTM, and a novel Intervention Interaction Attention Module (IIAM) for joint prediction of seven ICU interventions. FIRST-ICU was developed using MIMIC-IV (*n* = 23,926) and externally validated on AmsterdamUMCdb (*n* = 12,603) without retraining. The Temporal Decoder achieved AUC-ROC > 0.98 for all interventions with Brier scores <0.035. The Discrete Decoder outperformed state-of-the-art baselines, achieving the highest Macro Average Precision for six of seven interventions. Gains were largest for vasopressor interventions, with norepinephrine and phenylephrine improving by 28.0% and 32.8%, respectively, over CNN baselines. Ablation analysis showed that IIAM improved AUC-PR, particularly for vasopressor prediction. Cold-start analysis confirmed robust prediction from physiological signals alone (AUC-PR 0.667–0.868). External validation maintained AUC-ROC > 0.90 across intervention categories despite substantial differences in prescribing practices. A Generative Topographic Mapping layer identified six clinically distinct phenotypes, enabling interpretable risk stratification. FIRST-ICU advances multi-intervention prediction through joint modelling of treatment co-occurrence patterns, validated physiological learning, cross-continental generalisability, and interpretable risk stratification. It provides a framework for multi-intervention ICU decision support.

## Introduction

Intensive Care Units (ICUs) represent the frontline of modern medicine, providing life-sustaining interventions for patients with acute organ failures and life-threatening conditions. The complexity of ICU care has escalated substantially over recent decades, driven by an aging population with multiple comorbidities, advances in life-support technologies, and increasing survival from previously fatal conditions^1^. Managing critically ill patients presents unique challenges: physiological instability evolves rapidly, often across multiple organ systems simultaneously; deterioration can occur within minutes to hours; and therapeutic decisions must balance the competing risks of under-treatment and iatrogenic harm^2,3^. Clinicians must continuously integrate high-dimensional, multimodal data streams (vital signs, laboratory values, imaging findings, and treatment responses) to guide interventions such as mechanical ventilation, vasopressor support, and renal replacement therapy^4^. This cognitive burden is compounded by the temporal interdependencies between interventions, where the need for one therapy (e.g. sedation for agitation) may precipitate the need for another (e.g. mechanical ventilation for airway protection), creating complex decision cascades that challenge even experienced intensivists^5^.

The widespread adoption of electronic health records (EHRs) has transformed ICU data from ephemeral bedside observations into comprehensive digital archives, creating unprecedented opportunities for data-driven clinical decision support^6,7^. Modern EHRs capture high-resolution physiological time series, medication administrations, laboratory results, and procedural interventions at scale. Publicly available critical care databases such as MIMIC-III, MIMIC-IV, eICU and AmsterdamUMCdb have catalysed research by providing investigators access to de-identified data from hundreds of thousands of ICU admissions^8–10^. However, ICU data present unique analytical challenges: high dimensionality with hundreds of variables per patient, temporal irregularity with variable sampling rates across different measurements, pervasive missingness due to clinical decision-making about test ordering, measurement noise from sensor artifacts and patient movement, and complex censoring patterns from competing events^11–13^. Successfully leveraging these data requires sophisticated analytical frameworks capable of handling these characteristics while extracting clinically meaningful patterns that can inform timely interventions and improve patient outcomes^14^.

Recent advances in machine learning, particularly deep learning (DL), have shown considerable promise for modelling ICU data, owing to their ability to learn hierarchical representations from raw, heterogeneous inputs without extensive feature engineering^14–16^. Recurrent architectures such as long short-term memory (LSTM) networks and gated recurrent units (GRUs) can capture temporal dependencies in irregular time series while maintaining memory of distant past events^17,18^. Convolutional approaches have proven effective for extracting local temporal patterns and multi-scale features^19,20^, while attention mechanisms enable models to identify clinically relevant time points and focus computational resources on the most informative measurements^21^. More recently, graph neural networks (GNNs) have emerged as a natural framework for modelling the complex interdependencies between physiological variables and interventions, representing clinical data as graphs where nodes correspond to measurements and edges encode their relationships^22,23^. Despite these methodological advances, translating predictive models into clinical practice remains challenging, requiring not only high discrimination performance but also appropriate calibration, interpretability, generalisability across institutions, and alignment with clinical workflows and decision-making timescales^24,25^.

A substantial body of work has applied machine learning to predict ICU interventions, motivated by the potential to provide early warnings that enable proactive rather than reactive care. Early approaches focused on predicting single interventions in isolation. Zeng et al.^26^. utilised LSTM and gated recurrent neural networks to predict extubation failure risk in mechanically ventilated ICU patients, while Kwak et al.^27^. developed a bidirectional LSTM model to predict the need for vasopressors within the first 24 h of ICU admission. Similarly, Ghassemi et al.^28^. trained unsupervised switching state autoregressive models on vital signs to predict the onset of five ICU interventions, including mechanical ventilation, vasopressor administration, and three types of transfusions (packed red blood cells, fresh frozen plasma, and platelets). While demonstrating the feasibility of intervention prediction, these studies were limited by their focus on predicting only the initiation of single interventions, without modelling ongoing treatment trajectories or the relationships between concurrent therapies.

A more sophisticated approach was proposed by Suresh et al.^29^, which used deep learning to predict medical interventions within 4-hour windows following a 6-h observation period. Their framework employed both LSTM networks and convolutional neural networks (CNN), training separate models for five interventions: invasive mechanical ventilation, non-invasive ventilation, vasopressors, colloid boluses, and crystalloid boluses. Each intervention was classified into four discrete states: onset, wean, stay on, and stay off, capturing both initiation and discontinuation of therapies. Incorporating unstructured clinical notes via topic modelling further improved prediction performance, particularly for less invasive interventions. Xu et al.^22^ extended this approach with a graph convolutional neural network (GCNN) that explicitly models dependencies between clinical variables. Their architecture combines temporal convolution modules for time-domain representations with graph convolution modules for physiological relationships, and they applied the same discrete state framework as Suresh et al. to mechanical ventilation and vasopressors. This approach achieved substantial improvements over baseline methods, while interpretability analysis revealed physiologically meaningful relationships, such as connections between blood pressure and vasopressor administration. They preferred discrete states over hourly labels to avoid impulsive training signals and category imbalance, where switch states are much less frequent than keep states. While this demonstrates that graph-based architectures can capture clinically relevant dependencies, these methods still model interventions independently and do not account for multiple concurrent therapies or continuous intervention trajectories. More recently, transformer-based architectures and attention mechanisms have been applied to ICU time series modelling^21,30^, demonstrating improvements in capturing long-range temporal dependencies and handling irregular sampling patterns.

However, a fundamental limitation persists across existing work: the predominant focus on single-intervention prediction with separate models trained independently for each target. This approach has several critical shortcomings that limit both its clinical utility and scientific rigour. One major issue is that it ignores the clinical reality that ICU patients commonly require multiple simultaneous interventions. A patient on mechanical ventilation often requires sedation and analgesia, may need vasopressor support for hemodynamic instability, could require renal replacement therapy for acute kidney injury, and might receive multiple fluid resuscitations, with complex dependencies between these therapeutic decisions^31,32^. Another limitation is that training independent models fails to leverage shared representations and the sample efficiency benefits of multi-task learning, which has been shown to improve generalisation by enabling models to learn common underlying patterns across related prediction tasks^33–35^. A further concern is that existing approaches do not explicitly model the co-dependencies between interventions themselves. For example, the initiation of mechanical ventilation often correlates with increased sedation requirements, while aggressive fluid resuscitation may necessitate subsequent vasopressor support, yet these relationships are not captured when interventions are predicted independently. Additionally, discretising continuous intervention trajectories into fixed categorical states within rigid prediction windows discards valuable information about the precise timing and sequence of changes, potentially limiting utility for real-time clinical decision support. Finally, most existing work has focused on single-centre validation, raising questions about generalisability across institutions with different patient populations, clinical practices and documentation patterns^36^.

In this work, we introduce FIRST-ICU (Forecasting Interventions and Risk Stratification in the ICU), a unified deep learning framework that addresses these limitations through joint modelling of multiple ICU interventions with explicit attention to treatment co-occurrence patterns. Our approach simultaneously predicts seven clinically relevant interventions reflecting the spectrum of respiratory and hemodynamic support in critical care: non-invasive ventilation, invasive mechanical ventilation^37^, and five vasoactive medications (norepinephrine, phenylephrine, dopamine, epinephrine, and dobutamine)^38,39^. By modelling these interventions jointly rather than independently, FIRST-ICU captures the complex interplay between respiratory failure and circulatory shock, two of the most common and life-threatening conditions in the ICU^40,41^.

Our key contributions are as follows: (1) We develop a unified multi-task learning framework that simultaneously forecasts multiple interventions, capturing statistical co-occurrence patterns that are missed when treatments are modelled independently, reflecting clinical reality where patients often require concurrent therapies with complex interdependencies. (2) We introduce architectural mechanisms that capture statistical intervention co-occurrence patterns, enabling the model to learn associations such as the common co-administration of multiple vasopressors in refractory shock or the progression from non-invasive to invasive ventilation in respiratory failure. (3) We provide dual prediction paradigms: categorical state predictions (onset, wean, stay on, stay off) enabling direct comparison with existing methods, and continuous hourly probability forecasts over a 6-hour horizon aligned with clinical decision-making timescales. (4) Through focused cold-start analysis of onset prediction cases where no prior intervention history exists, we address concerns about target leakage and validate that the model captures true early warning signals from physiological deterioration rather than simply extrapolating recent treatment patterns. (5) We establish cross-continental generalisability by validating on an independent European cohort (AmsterdamUMCdb), focusing on physiological needs (respiratory failure, shock states) rather than institution-specific drug choices, demonstrating that learned patterns transfer across healthcare systems. (6) We incorporate unsupervised clustering that projects patients into an interpretable latent space, revealing distinct phenotypes associated with different clinical outcomes and intervention trajectories.

**Fig. 1:**
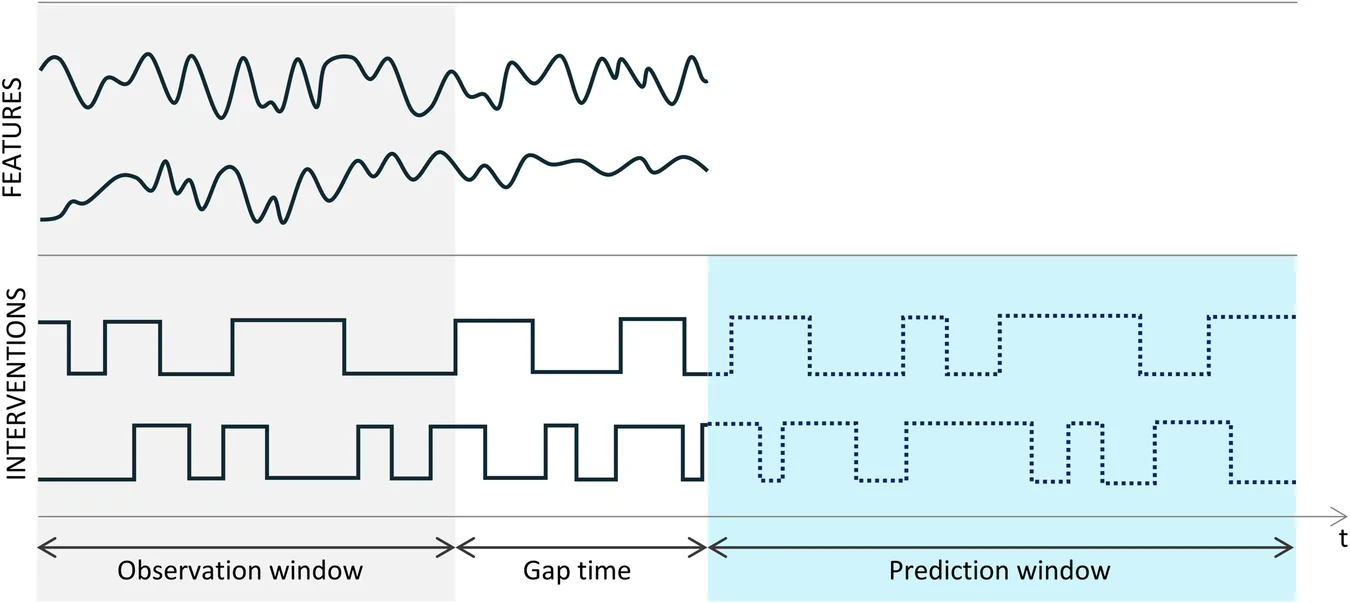
FIRST-ICU temporal window structure for multi-intervention forecasting*.* The framework uses a 12-h observation window (*T* = 1 to *T* = 12) containing patient features including vital signs, laboratory values, and intervention history. A 6-h gap period (*T* = 13 to *T* = 18) separates observation from prediction, allowing identification of early warning signals before intervention need. The prediction window spans *T* = 19 to *T* = 24 (6 h for the Temporal Decoder) or *T* = 19 to *T* = 22 (4 h for the Discrete Decoder, following Suresh et al.^29^). Solid coloured boxes represent observed interventions in the observation window; dashed boxes with gradient shading represent hourly probability predictions. Multiple interventions are shown to illustrate the multi-task prediction paradigm.

**Fig. 2:**
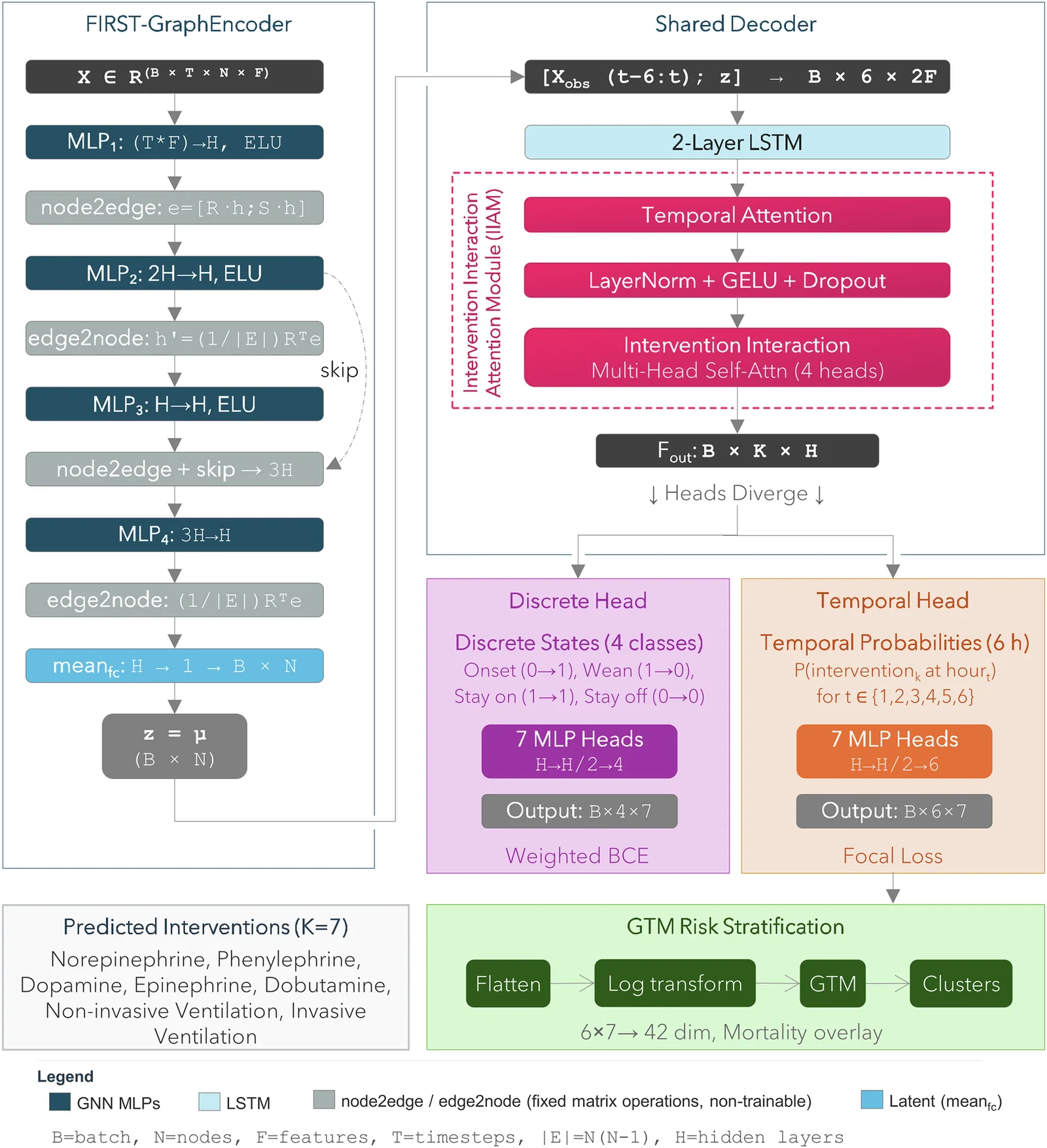
FIRST-ICU graph neural network autoencoder (GNN-AE) architecture. The model consists of three main components: (1) FIRST-GraphEncoder: A graph neural network encoder that transforms multivariate clinical time series (X ∈ ℝ^{B×T×N×F}) into latent representations through two rounds of message passing. Dark blue boxes indicate trainable MLPs; light-grey boxes indicate non-trainable graph aggregation operations. (2) Shared Decoder: Processes latent representations through a 2-layer LSTM (hidden size H) followed by the intervention interaction attention module (IIAM, highlighted in the dashed box). The IIAM comprises temporal attention (identifying informative timesteps), shared feature encoder (layer normalisation + GELU + dropout), and intervention interaction layer (4-head self-attention enabling interventions to attend to each other). (3) Prediction Heads: Two interchangeable decoder variants: the Discrete head outputs SoftMax probabilities over four categorical states (Onset, Wean, Stay on, Stay off) per intervention; the Temporal head outputs sigmoid probabilities for each hour in the 6-h prediction horizon per intervention. GTM Risk Stratification operates on Temporal Decoder outputs for unsupervised patient phenotyping.

We demonstrate that FIRST-ICU outperforms state-of-the-art single-task methods across multiple metrics while providing interpretable risk stratification and maintaining performance in external validation. Figure 1 illustrates the temporal prediction setup and target formulation, while Fig. 2 summarises the overall FIRST-ICU architecture. The combination of multi-intervention forecasting, validated physiological learning, and cross-continental generalisability positions FIRST-ICU as a comprehensive framework for ICU decision support.

## Results

### Cohort characteristics

The primary development and internal evaluation cohort was derived from MIMIC-IV and comprised 23,926 ICU admissions after applying the inclusion and exclusion criteria described in ‘Data extraction and preprocessing’. The median age was 67.0 years (IQR 56.0–78.0), with 58.83% male patients (Table 1). Invasive ventilation was administered in 63.82% of admissions, while norepinephrine was the most frequently used vasopressor (28.71%), followed by phenylephrine (37.37%).

**Table 1:** Baseline characteristics of study cohorts

Variables	MIMIC-IV Median [Q1, Q3]	MIMIC-IV [Min, Max]/Proportion	Amsterdam Median [Q1, Q3]	Amsterdam [Min, Max]/Proportion
Demographics				
Age [years]	67.0 [56.0, 78.0]	[18.0, 89.0]	64.5 [54.5, 74.5]	[28.5, 85.0]
Sex [male]	-	58.83%	-	62.94%
Weight [kg]	80.0 [67.2, 94.2]	[0.0, 296.8]	NA	NA
Height [cm]	170.0 [167.0, 172.1]	[58.7, 215.2]	174.5 [164.5, 184.5]	[164.5, 195.0]
Ethnicity:				
Asian [yes]	-	2.81%	NA	NA
Black [yes]	-	8.40%	NA	NA
Hispanic [yes]	-	3.36%	NA	NA
White [yes]	-	66.77%	NA	NA
Other [yes]	-	18.65%	NA	NA
Vitals:				
Temperature [°C]	36.9 [36.7, 37.1]	[31.6, 39.7]	36.7 [36.3, 37.1]	[23.0, 40.1]
Capillary refill [yes]	-	14.23%	NA	NA
Diastolic blood pressure [mmHg]	60.0 [54.0, 67.0]	[28.0, 116.0]	61.0 [56.0, 67.6]	[29.0, 118.7]
Fraction inspired oxygen [%]	40.0 [21.0, 50.0]	[21.0, 100.0]	40.5 [40.0, 46.0]	[21.0, 100.0]
Glasgow Coma Scale (GCS) eye opening	4.0 [3.0, 4.0]	[1.0, 4.0]	4.0 [3.0, 4.0]	[1.0, 4.0]
Glasgow Coma Scale (GCS) motor response	6.0 [6.0, 6.0]	[1.0, 6.0]	6.0 [5.0, 6.0]	[1.0, 6.0]
Glasgow Coma Scale (GCS) verbal response	5.0 [1.0, 5.0]	[1.0, 5.0]	4.0 [1.0, 5.0]	[1.0, 5.0]
Glucose [mg/dL]	128.0 [112.0, 150.0]	[51.0, 817.0]	131.5 [120.7, 149.6]	[45.0, 474.8]
Haemoglobin [g/dL]	9.7 [8.7, 11.1]	[3.6, 19.9]	10.5 [9.5, 11.8]	[0.6, 49.9]
Heart rate [beats per min]	84.2 [76.0, 94.0]	[26.0, 162.0]	83.0 [74.0, 93.0]	[35.5, 159.0]
Magnesium [mg/dL]	2.1 [1.9, 2.3]	[0.8, 6.4]	2.1 [1.9, 2.4]	[0.8, 7.6]
Mean blood pressure	75.8 [70.0, 83.0]	[35.0, 127.0]	83.0 [76.0, 90.0]	[14.0, 135.5]
Blood pH Level	7.4 [7.4, 7.4]	[6.7, 7.8]	7.4 [7.4, 7.4]	[6.6, 7.8]
Platelet count [k/uL]	169.0 [120.0, 236.0]	[5.0, 1153.0]	191.0 [138.0, 265.0]	[1.0, 1216.0]
Prothrombin time [s]	13.7 [12.3, 15.7]	[7.6, 98.8]	NA	NA
Respiratory rate [breaths per min]	19.0 [17.0, 22.0]	[6.0, 40.0]	18.0 [15.5, 21.7]	[5.3, 77.0]
Oxygen saturation [%]	97.0 [96.0, 98.0]	[54.0, 100.0]	97.0 [95.4, 98.0]	[42.0, 100.0]
Systolic blood pressure [mmHg]	116.0 [107.0, 127.0]	[48.0, 191.5]	126.7 [115.0, 139.0]	[46.0, 210.8]
Anion Gap [mmol/L]	13.0 [11.0, 15.0]	[3.0, 52.0]	8.6 [6.8, 10.5]	[4.9, 42.8]
Lactate	1.6 [1.2, 2.2]	[0.5, 25.1]	1.4 [1.0, 2.1]	[0.5, 25.2]
Phosphate	3.3 [2.7, 3.9]	[0.5, 14.5]	3.3 [2.7, 4.0]	[0.3, 13.6]
Creatinine	1.0 [0.7, 1.5]	[0.2, 22.1]	0.9 [0.7, 1.3]	[0.2, 17.7]
Procedures				
Non-invasive ventilation [yes]	-	4.86%	-	10.28%
Invasive ventilation [yes]	-	63.82%	-	77.20%
Drugs				
Dobutamine [yes]	-	2.01%	-	9.60%
Norepinephrine [yes]	-	28.71%	-	56.76%
Phenylephrine [yes]	-	37.37%	-	0.23%
Dopamine [yes]	-	3.66%	-	27.49%
Epinephrine [yes]	-	8.19%	-	3.16%
Heart rhythms				
Atrial fibrillation [yes]	-	25.33%	-	27.34%
Atrial flutter [yes]	-	5.88%	-	5.59%
1st Degree atrioventricular block [yes]	-	8.14%	-	7.15%
2nd or 3rd degree atrioventricular block [yes]	-	1.35%	-	2.16%
Asystole (absence of ventricular activity) [yes]	-	1.46%	-	1.04%
Bundle branch block (BBB) [yes]	-	3.23%	-	0.42%
Junctional rhythm or junctional tachycardia (JR-JT) [yes]	-	3.28%	-	4.45%
Supraventricular tachycardia (SVT) [yes]	-	4.41%	-	7.63%
Ventricular tachycardia or ventricular fibrillation (VT-VF) [yes]	-	1.47%	-	8.57%

Continuous variables are reported as median [Q1, Q3] with [min, max] range; categorical variables as prevalence (%). Variables unavailable in AmsterdamUMCdb are indicated as NA. The MIMIC-IV cohort (*n* = 23,926) was used for model development and internal validation; the AmsterdamUMCdb cohort (*n* = 12,603) was used for external validation without model retraining.

The external validation cohort was obtained from the AmsterdamUMCdb critical care database and comprised 12,603 ICU admissions. This European cohort exhibited higher intervention prevalence: invasive ventilation in 77.20% of admissions and norepinephrine in 56.76% (Table 1). Notable differences in drug utilisation patterns between cohorts included substantially lower phenylephrine use in AmsterdamUMCdb (0.23% vs 37.37% in MIMIC-IV) and higher dopamine use (27.49% vs 3.66%), consistent with regional differences in prescribing practices reported in prior studies^42,43^.

Because several variables available in MIMIC-IV were not recorded in AmsterdamUMCdb, including ethnicity, capillary refill time, patient weight, and prothrombin time, the model was retrained on MIMIC-IV using only the subset of features shared between the two databases. This harmonisation ensured that external validation performance differences reflect true domain shift rather than discrepancies in feature availability.

### Internal validation on MIMIC-IV

For the Discrete Decoder, the prediction targets exhibited severe class imbalance across all seven interventions (Table 2). The STAY OFF state dominated for most interventions, ranging from 53.4% for invasive ventilation to 99.1% for dopamine. Transition states (ONSET and WEAN) were rare: invasive ventilation ONSET occurred in only 0.4% of prediction windows, while vasopressor ONSET events ranged from 0.1% (dobutamine) to 1.8% (norepinephrine). Given this class imbalance, Macro AUC-PR was used as the primary evaluation metric.

**Table 2:** Class prevalence for discrete intervention state prediction

Interventions	Stay off	Stay on	Wean	Onset
Invasive ventilation	0.534	0.450	0.012	0.004
Non-Invasive ventilation	0.990	0.008	0.001	0.001
Norepinephrine	0.868	0.090	0.024	0.018
Phenylephrine	0.926	0.047	0.015	0.012
Dobutamine	0.990	0.008	0.001	0.001
Dopamine	0.991	0.006	0.001	0.002
Epinephrine	0.988	0.010	0.001	0.001

Values represent the proportion of 4-h prediction windows assigned to each categorical state. STAY OFF and STAY ON indicate stable intervention status; ONSET and WEAN indicate transitions during the prediction window. The severe imbalance, particularly for transition states (<2% for most interventions), motivates the use of precision-recall metrics and class-weighted loss functions.

The Discrete Decoder was evaluated against three state-of-the-art baseline methods (Suresh et al.^29^ LSTM and CNN, Xu et al.^22^ GCNN) using Macro Average Precision (AP), Macro AUC-PR, and Macro F1 (Table 3). All methods were evaluated on identical test sets with 1000 bootstrap iterations for confidence interval estimation. FIRST-ICU achieved the highest Macro AP for six of seven interventions, the highest Macro F1 for six of seven interventions, and the highest Macro AUC-PR for five of seven interventions.

**Table 3:** Comparison of FIRST-ICU Discrete Decoder with baseline methods across all seven interventions

Intervention	Metric	FIRST-ICU	CNN^29^	LSTM^29^	GCNN^22^
Invasive ventilation	Macro AP	**0.5159 (0.5007–0.5375)**	0.4809 (0.4774–0.4877)	0.4853 (0.4813–0.4930)	0.5046 (0.4960–0.5260)
	Macro AUC-PR	**0.5140 (0.4992–0.5344)**	0.4802 (0.4765–0.4851)	0.4843 (0.4801–0.4898)	0.5004 (0.4944–0.5184)
	Macro F1	**0.5121 (0.4866–0.5407)**	0.4602 (0.4487–0.4727)	0.4704 (0.4524–0.4815)	0.4738 (0.4717–0.5004)
Non-invasive ventilation	Macro AP	**0.5186 (0.4253–0.6289)**	0.4046 (0.3628–0.4875)	0.5176 (0.4588–0.6213)	0.4025 (0.3774–0.4365)
	Macro AUC-PR	**0.5067 (0.4067–0.6032)**	0.3808 (0.3436–0.4538)	0.4887 (0.4314–0.5710)	0.4508 (0.4053–0.4990)
	Macro F1	**0.5188 (0.3862–0.6817)**	0.4212 (0.2971–0.4648)	0.4466 (0.3899–0.5612)	0.2580 (0.2491–0.4398)
Norepinephrine	Macro AP	**0.4535 (0.4439–0.4648)**	0.3543 (0.3475–0.3642)	0.3528 (0.3462–0.3628)	0.4509 (0.4438–0.4613)
	Macro AUC-PR	**0.4528 (0.4423–0.4632)**	0.3536 (0.3462–0.3626)	0.3517 (0.3446–0.3607)	0.4493 (0.4414–0.4583)
	Macro F1	**0.4551 (0.4278–0.4823)**	0.3789 (0.3350–0.3920)	0.3523 (0.3277–0.3852)	0.4336 (0.2971–0.4660)
Phenylephrine	Macro AP	**0.4143 (0.4011–0.4315)**	0.3125 (0.3057–0.3229)	0.3026 (0.2973–0.3131)	0.4099 (0.4000–0.4229)
	Macro AUC-PR	**0.4125 (0.3987–0.4276)**	0.3114 (0.3041–0.3205)	0.3013 (0.2955–0.3096)	0.4087 (0.3980–0.4211)
	Macro F1	**0.4296 (0.4014–0.4450)**	0.2985 (0.2615-0.3138)	0.2565 (0.2446–0.3170)	0.4271 (0.2912–0.4363)
Dobutamine	Macro AP	0.4541 (0.4274–0.5138)	0.4646 (0.4447–0.4933)	**0.4771 (0.4479–0.5482)**	0.4572 (0.4335–0.5015)
	Macro AUC-PR	0.4428 (0.4169–0.4799)	0.4596 (0.4366–0.4792)	0.4674 (0.4395–0.5055)	**0.5391 (0.4860–0.5908)**
	Macro F1	0.4536 (0.3596–0.4690)	0.4410 (0.3929–0.4812)	**0.4803 (0.4230–0.5551)**	0.4536 (0.2495–0.5229)
Dopamine	Macro AP	**0.4267 (0.3977–0.4767)**	0.3992 (0.3691–0.4401)	0.3909 (0.3632–0.4391)	0.4105 (0.3839–0.4464)
	Macro AUC-PR	0.4184 (0.3866–0.4547)	0.3933 (0.3593–0.4277)	0.3849 (0.3510–0.4213)	**0.4883 (0.4426–0.5331)**
	Macro F1	**0.4595 (0.3644–0.5203)**	0.3959 (0.3509–0.4402)	0.3641 (0.3031–0.3938)	0.4194 (0.2639–0.5079)
Epinephrine	Macro AP	**0.4393 (0.4147–0.4818)**	0.4057 (0.3714–0.4867)	0.3798 (0.3504–0.4481)	0.4182 (0.3950–0.4686)
	Macro AUC-PR	**0.4333 (0.4075–0.4616)**	0.3982 (0.3626–0.4408)	0.3727 (0.3417–0.4110)	0.4079 (0.3825–0.4382)
	Macro F1	**0.5179 (0.3800–0.5762)**	0.3935 (0.3373–0.4483)	0.3832 (0.2780–0.4498)	0.4144 (0.3991–0.4452)

Metrics: Macro Average Precision (AP), Macro AUC-PR, and Macro F1 with 95% confidence intervals from 1000 bootstrap iterations. All methods were trained using weighted cross-entropy loss with identical class weights. CNN and LSTM baselines follow Suresh et al.^29^; GCNN follows Xu et al.^22^. Bold values indicate highest performance per intervention-metric combination. FIRST-ICU outperforms baselines for 5/7 interventions on Macro AUC-PR.

For the four higher-prevalence interventions (invasive ventilation, non-invasive ventilation, norepinephrine, and phenylephrine), FIRST-ICU achieved the highest values across all three metrics (Table 3). The largest performance differences were observed for vasopressor interventions: for norepinephrine, FIRST-ICU achieved Macro AP of 0.454 compared to 0.354 for CNN, a relative improvement of 28.2%. For phenylephrine, FIRST-ICU achieved Macro AP of 0.414 compared to 0.313 for CNN, a relative improvement of 32.3%. Statistically significant improvements (non-overlapping 95% confidence intervals) were observed for FIRST-ICU over all baselines for invasive ventilation.

For low-prevalence interventions, performance patterns varied across metrics (Table 3). For epinephrine, FIRST-ICU achieved the highest values on all three metrics. For dopamine, GCNN achieved the highest Macro AUC-PR (0.488), while FIRST-ICU achieved the highest Macro AP (0.427) and Macro F1 (0.460). For dobutamine, GCNN achieved the highest Macro AUC-PR (0.539) but the lowest Macro AP (0.457) among all methods; LSTM achieved the highest Macro AP (0.477) and Macro F1 (0.480). Confidence intervals for low-prevalence interventions were wider due to limited sample sizes (prevalence below 1%).

To contextualise computational cost, we report parameter counts for all compared models: FIRST-ICU (Discrete Decoder) contains 1,792,015 parameters, compared to 3,239,940 for the LSTM baseline, 229,572 for the CNN baseline, and 34,708 for the GCNN. FIRST-ICU achieves superior performance with approximately half the parameters of the LSTM baseline, suggesting that improvements arise from architectural design rather than increased model capacity.

For the temporal decoder, performance was evaluated on the MIMIC-IV test set using AUC-ROC, AUC-PR, and Brier score for each intervention across the 6-h prediction horizon (Table 4). Performance metrics are reported for each hour from *T* = 19 to *T* = 24, corresponding to hours 1–6 in the prediction window following the 12-h observation period and 6-h gap.

**Table 4:** Temporal decoder performance on MIMIC-IV internal validation

Intervention	Metric	*T* = 19	*T* = 20	*T* = 21	*T* = 22	*T* = 23	*T* = 24
Invasive ventilation	*AUC-ROC*	0.9957 (0.9948–0.9964)	0.9954 (0.9945–0.9963)	0.9956 (0.9948–0.9964)	0.9950 (0.9939–0.9959)	0.9947 (0.9938–0.9957)	0.9940 (0.9929–0.9950)
	*AUC-PR*	0.9949 (0.9939–0.9958)	0.9945 (0.9933–0.9956)	0.9944 (0.9927–0.9957)	0.9928 (0.9905–0.9947)	0.9928 (0.9908–0.9945)	0.9913 (0.9889–0.9933)
	*Brier Score*	0.0258 (0.0238–0.0278)	0.0258 (0.0237–0.0279)	0.0260 (0.0239–0.0282)	0.0267 (0.0246–0.0288)	0.0283 (0.0262–0.0304)	0.0309 (0.0288–0.0330)
	*Prevalence*	46.23%	45.89%	45.59%	45.46%	45.17%	45.02%
Non-invasive ventilation	*AUC-ROC*	0.9882 (0.9728–0.9979)	0.9794 (0.9536–0.9975)	0.9887 (0.9719–0.9984)	0.9898 (0.9734–0.9991)	0.9887 (0.9722–0.9981)	0.9896 (0.9716–0.9986)
	*AUC-PR*	0.8642 (0.8031–0.9198)	0.8609 (0.7970–0.9137)	0.8678 (0.8046–0.9218)	0.8950 (0.8358–0.9409)	0.8742 (0.8104–0.9269)	0.8784 (0.8214–0.9293)
	*Brier score*	0.0034 (0.0027–0.0042)	0.0034 (0.0026–0.0042)	0.0031 (0.0024–0.0039)	0.0028 (0.0022–0.0035)	0.0032 (0.0025–0.0039)	0.0035 (0.0028–0.0042)
	*Prevalence*	0.92%	0.96%	0.92%	0.92%	0.92%	0.90%
Norepinephrine	*AUC-ROC*	0.9857 (0.9835–0.9878)	0.9864 (0.9843–0.9884)	0.9870 (0.9849–0.9889)	0.9869 (0.9850–0.9890)	0.9871 (0.9851–0.9888)	0.9851 (0.9824–0.9874)
	*AUC-PR*	0.8753 (0.8543–0.8954)	0.8842 (0.8663–0.9017)	0.8894 (0.8711–0.9065)	0.8960 (0.8774–0.9123)	0.8936 (0.8760–0.9101)	0.8803 (0.8592–0.8994)
	*Brier Score*	0.0324 (0.0304–0.0345)	0.0305 (0.0286–0.0325)	0.0295 (0.0275–0.0313)	0.0294 (0.0275–0.0313)	0.0302 (0.0283–0.0321)	0.0325 (0.0304–0.0344)
	*Prevalence*	11.33%	11.26%	11.21%	11.13%	10.98%	10.95%
Phenylephrine	*AUC-ROC*	0.9852 (0.9817–0.9883)	0.9846 (0.9804–0.9879)	0.9860 (0.9823–0.9888)	0.9854 (0.9817-0.9889)	0.9855 (0.9818–0.9888)	0.9846 (0.9804–0.9879)
	*AUC-PR*	0.8328 (0.8026–0.8620)	0.8263 (0.7956–0.8552)	0.8265 (0.7962–0.8571)	0.8461 (0.8189–0.8728)	0.8335 (0.8036–0.8615)	0.8164 (0.7806–0.8473)
	*Brier Score*	0.0256 (0.0238–0.0272)	0.0242 (0.0225–0.0259)	0.0231 (0.0215–0.0248)	0.0227 (0.0210–0.0243)	0.0232 (0.0216–0.0247)	0.0250 (0.0233–0.0266)
	*Prevalence*	6.19%	6.14%	6.07%	6.07%	5.97%	5.74%
Dobutamine	*AUC-ROC*	0.9990 (0.9973–0.9999)	0.9992 (0.9977–0.9999)	0.9992 (0.9980–0.9999)	0.9993 (0.9982–0.9999)	0.9993 (0.9981–0.9999)	0.9992 (0.9979–0.9999)
	*AUC-PR*	0.9666 (0.9355–0.9897)	0.9667 (0.9336–0.9894)	0.9656 (0.9335–0.9895)	0.9629 (0.9333–0.9873)	0.9651 (0.9354–0.9878)	0.9567 (0.9198–0.9830)
	*Brier Score*	0.0014 (0.0010–0.0018)	0.0014 (0.0010–0.0019)	0.0014 (0.0010-–	0.0015 (0.0010–0.0020)	0.0015 (0.0010-–	0.0016 (0.0012–0.0022)
	*Prevalence*	0.90%	0.87%	0.88%	0.89%	0.86%	0.83%
Dopamine	*AUC-ROC*	0.9990 (0.9980–0.9997)	0.9988 (0.9978–0.9996)	0.9990 (0.9980–0.9996)	0.9990 (0.9981–0.9997)	0.9990 (0.9980–0.9997)	0.9988 (0.9978–0.9995)
	*AUC-PR*	0.9194 (0.8622–0.9623)	0.9180 (0.8633–0.9610)	0.9196 (0.8645–0.9600)	0.9286 (0.8814–0.9654)	0.9272 (0.8743–0.9688)	0.9073 (0.8490–0.9553)
	*Brier Score*	0.0021 (0.0016–0.0027)	0.0022 (0.0017–0.0028)	0.0020 (0.0015–0.0025)	0.0018 (0.0013–0.0023)	0.0019 (0.0014–0.0025)	0.0021 (0.0016–0.0028)
	*Prevalence*	0.71%	0.67%	0.74%	0.77%	0.73%	0.72%
Epinephrine	*AUC-ROC*	0.9987 (0.9980–0.9994)	0.9988 (0.9980–0.9994)	0.9987 (0.9978–0.9993)	0.9941 (0.9840–0.9991)	0.9930 (0.9828–0.9991)	0.9907 (0.9806–0.9987)
	*AUC-PR*	0.9199 (0.8736–0.9546)	0.9224 (0.8800–0.9584)	0.8993 (0.8397–0.9486)	0.8850 (0.8220–0.9356)	0.8841 (0.8223–0.9389)	0.8656 (0.7989–0.9235)
	*Brier score*	0.0034 (0.0027–0.0041)	0.0032 (0.0024–0.0038)	0.0033 (0.0025–0.0040)	0.0034 (0.0026–0.0042)	0.0032 (0.0025–0.0039)	0.0036 (0.0028–0.0043)
	*Prevalence*	1.11%	1.09%	1.03%	1.07%	1.11%	1.12%

Metrics: AUC-ROC (discrimination), AUC-PR (precision-recall under class imbalance), and Brier Score (calibration and accuracy) with 95% confidence intervals from 1000 bootstrap iterations. Performance is reported for each hour in the 6-hour prediction horizon (*T* = 19 to *T* = 24, corresponding to hours 1–6 ahead). Prevalence rows show the percentage of patients receiving each intervention at each timepoint. Higher AUC-ROC and AUC-PR indicate better performance; lower Brier scores indicate better calibration (perfect calibration = 0).

AUC-ROC exceeded 0.98 for all seven interventions at all prediction horizons (Table 4). For invasive ventilation, AUC-ROC ranged from 0.994 (95% CI: 0.993–0.995) at *T* = 24 to 0.996 (0.995–0.996) at *T* = 19. Dobutamine and dopamine achieved AUC-ROC exceeding 0.998 across all horizons. Invasive ventilation AUC-ROC decreased by 0.002 from *T* = 19 to *T* = 24.

AUC-PR varied by intervention prevalence (Table 4). For invasive ventilation (45–46% prevalence), AUC-PR exceeded 0.99 across all horizons: 0.995 (0.994–0.996) at *T* = 19 to 0.991 (0.989–0.993) at *T* = 24. For norepinephrine (11% prevalence), AUC-PR ranged from 0.875 to 0.896. For phenylephrine (6% prevalence), AUC-PR ranged from 0.816 to 0.846. For dobutamine, dopamine, and epinephrine (<1.5% prevalence), AUC-PR remained above 0.86.

Brier scores remained below 0.035 for all interventions across all prediction horizons (Table 4). For invasive ventilation, Brier scores ranged from 0.026 (0.024–0.028) at *T* = 19 to 0.031 (0.029–0.033) at *T* = 24. Dobutamine achieved 0.0014–0.0016, and dopamine achieved 0.0018–0.0022. Reliability diagrams are provided in Supplementary Fig. 1.

**Fig. 3:**
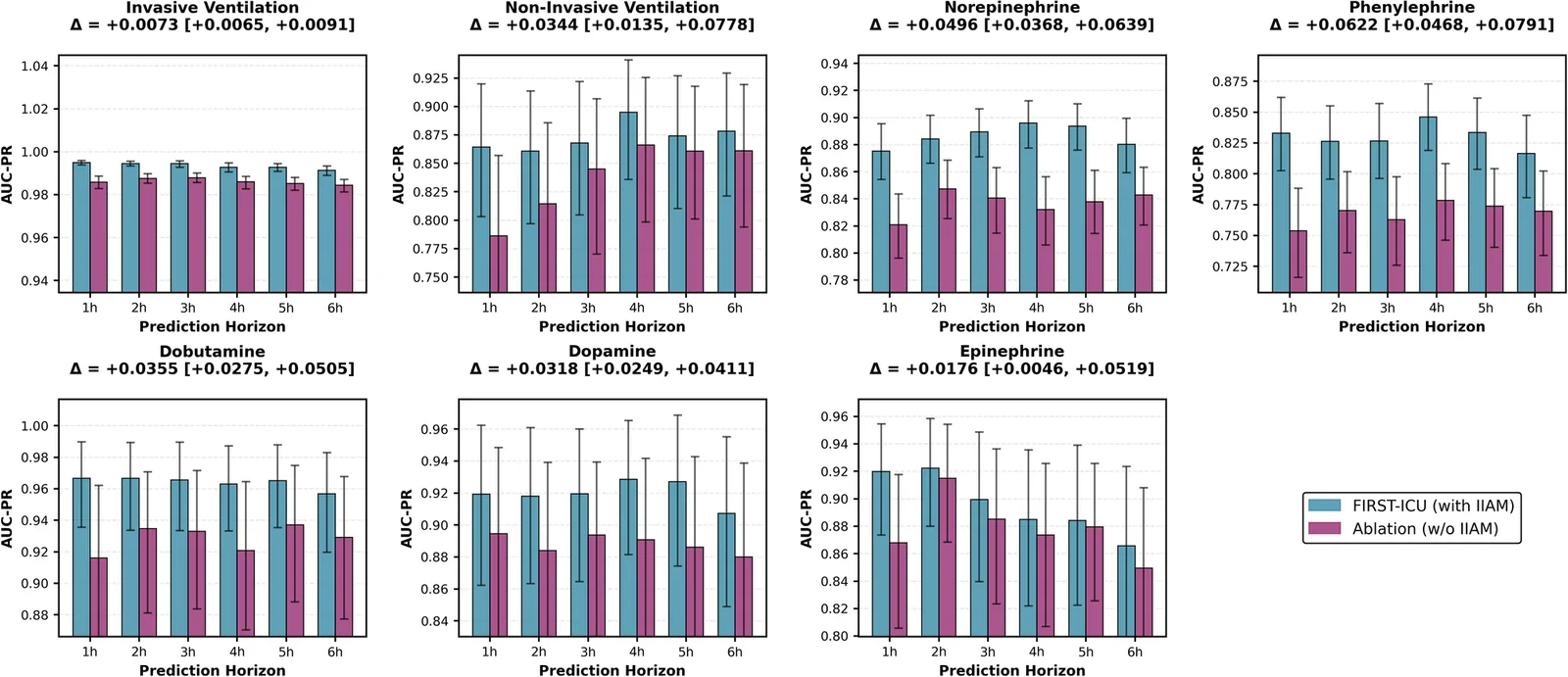
Ablation study: impact of the intervention interaction attention module (IIAM) on temporal decoder performance. Bar plots compare AUC-PR of the full model (with IIAM, blue) versus the ablated model (without IIAM, purple) across prediction horizons (1–6 h) for each intervention. Error bars show 95% confidence intervals from 1000 bootstrap iterations. Above each subplot, mean Δ indicates the average AUC-PR difference between models, while Δ range (min, max) shows variability across horizons. The shared legend in the eighth panel identifies the two model variants. Positive Δ values across all interventions confirm the IIAM consistently improves prediction performance, with largest gains observed for vasopressor interventions (norepinephrine, phenylephrine, epinephrine).

To quantify the contribution of the intervention interaction attention module (IIAM), we compared the full temporal decoder with an ablated variant in which LSTM outputs were passed directly to the prediction heads, bypassing temporal attention, shared encoding, and intervention interaction components (Fig. 3). Removing the IIAM resulted in consistent performance degradation across all interventions and prediction horizons.

For vasopressor interventions, the IIAM provided the largest performance gains (Fig. 3): phenylephrine showed mean ΔAUC-PR of +0.062 (range +0.047 to +0.079 across horizons), norepinephrine showed +0.050 (+0.037 to +0.064), and epinephrine showed +0.018 (+0.005 to +0.052). For ventilation interventions, gains were smaller: non-invasive ventilation +0.034 (+0.014 to +0.078) and invasive ventilation +0.007 (+0.007 to +0.009). For dobutamine, mean ΔAUC-PR was +0.031 (+0.019 to +0.054), and for dopamine +0.024 (+0.011 to +0.046).

Performance improvements were largest for phenylephrine and norepinephrine, and smallest for invasive ventilation. All seven interventions showed positive ΔAUC-PR across all six prediction horizons.

To assess performance in the clinically important cold-start setting, we evaluated patients with no intervention exposure during the 12-h observation window who subsequently required intervention during the 6-hour prediction horizon (Table 5).

**Table 5:** Cold-start onset prediction performance on MIMIC-IV internal validation

Intervention	*N*	*T* = 19	*T* = 20	*T* = 21	*T* = 22	*T* = 23	*T* = 24	Mean AUC-PR
Invasive ventilation	147	0.7535 (0.6656–0.8308)	0.8012 (0.7210-–	0.8402 (0.7725–0.9008)	0.8899 (0.8316–0.9353)	0.9435 (0.9032–0.9742)	0.9789 (0.9539–0.9948)	**0.8679**
Non-invasive ventilation	30	0.7820 (0.5651–0.9407)	0.8548 (0.6984–0.9589)	0.7629 (0.5569–0.9272)	0.8401 (0.6730–0.9531)	0.8204 (0.6435–0.9474)	0.8267 (0.6485–0.9576)	**0.8145**
Norepinephrine	183	0.6662 (0.5682–0.7689)	0.7602 (0.6713–0.8397)	0.8134 (0.7396–0.8778)	0.7971 (0.7259–0.8615)	0.8301 (0.7590–0.8928)	0.8489 (0.7859–0.9032)	**0.7860**
Phenylephrine	171	0.5433 (0.4354–	0.6030 (0.4925–0.7040)	0.6791 (0.5725–0.7793)	0.7013 (0.6032–0.7940)	0.7300 (0.6257–0.8269)	0.7444 (0.6426–0.8267)	**0.6669**
Dobutamine^a^	14	0.8859 (0.7029–1.0000)	0.7037 (0.3724–0.9419)	0.7049 (0.3713–0.9210)	0.7835 (0.5164–1.0000)	0.7247 (0.4449–0.9604)	0.7877 (0.5058–0.9890)	**0.7651**
Dopamine^a^	21	0.6602 (0.2875–0.9016)	0.6305 (0.2950–0.8696)	0.7582 (0.5279–0.9368)	0.8845 (0.7243–0.9876)	0.7356 (0.4556–0.9167)	0.7876 (0.5703–0.9374)	**0.7428**
Epinephrine^a^	22	0.6499 (0.3795–0.9116)	0.6548 (0.3865–0.9190)	0.6549 (0.3865–0.9190)	0.6933 (0.4444–0.9077)	0.9099 (0.7677–0.9956)	0.9763 (0.9094–1.0000)	**0.7565**

Cohort comprises patients with no intervention exposure during the 12-h observation window (*T* = 1–12) who required intervention during the 6-hour prediction horizon (*T* = 19–24). N indicates the number of cold-start cases per intervention; Mean Prevalence shows the average fraction with active intervention across the prediction horizon. AUC-PR values with 95% confidence intervals from 1000 bootstrap iterations.

^a^Interventions with *N* < 30 have limited statistical power.

Sample sizes varied by intervention: invasive ventilation *N* = 147, norepinephrine *N* = 183, phenylephrine *N* = 171 (Table 5). For rare interventions, sample sizes were limited: dobutamine *N* = 14, dopamine *N* = 21, epinephrine *N* = 22. Wide confidence intervals for these interventions reflect the limited sample sizes.

For invasive ventilation (*N* = 147, mean prevalence 75.74%), AUC-PR ranged from 0.754 (95% CI: 0.666–0.831) at *T* = 19–0.979 (0.954-0.995) at *T* = 24, with a mean AUC-PR of 0.868 across the prediction horizon (Table 5). For norepinephrine (*N* = 183, prevalence 63.11%), AUC-PR ranged from 0.666 (0.568–0.769) to 0.849 (0.786–0.903), with a mean of 0.786. For phenylephrine (*N* = 171, prevalence 56.63%), AUC-PR ranged from 0.543 (0.435–0.654) to 0.744 (0.643–0.827), with a mean of 0.667. AUC-PR increased across the prediction horizon for all interventions: invasive ventilation ΔAUC-PR + 0.225 (*T* = 19 to *T* = 24), norepinephrine +0.183, phenylephrine +0.201 (Table 5).

### External validation on AmsterdamUMCdb

**Fig. 4:**
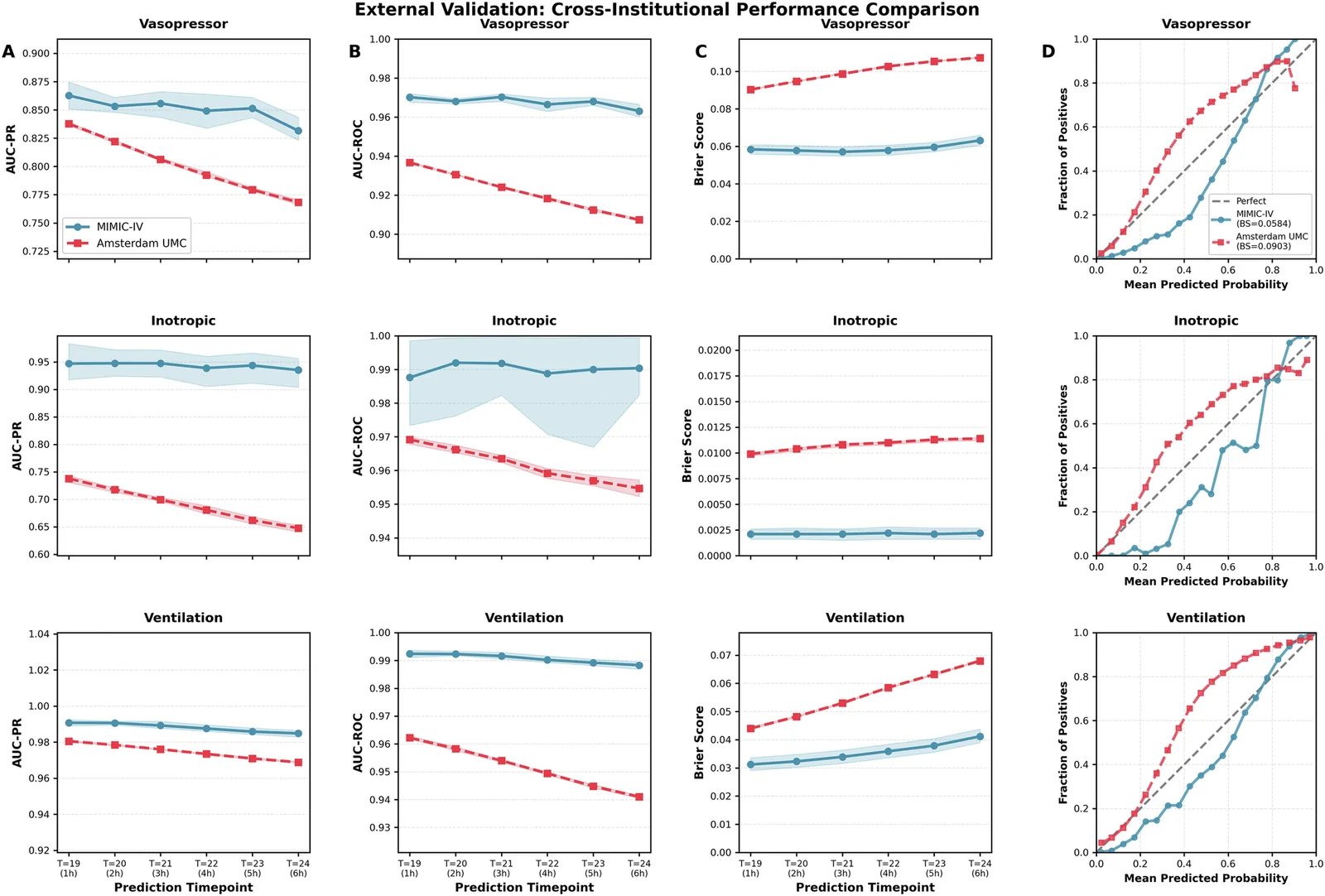
External validation: cross-institutional performance comparison between MIMIC-IV (internal validation) and AmsterdamUMCdb (external validation). **A** Macro AUC-PR, **B** Macro AUC-ROC, and **C** Brier Score across the 6-h prediction horizon (*T* = 19 to *T* = 24) for three intervention categories: vasopressor, inotropic, and ventilation. Blue solid lines represent MIMIC-IV; red dashed lines represent AmsterdamUMCdb. Shaded regions indicate 95% confidence intervals. **D** Calibration plots comparing both datasets; diagonal line represents perfect calibration.

External validation was first assessed for the Temporal Decoder on AmsterdamUMCdb, with predictions aggregated into three physiological need categories to accommodate differences in drug utilisation between cohorts (Fig. 4). External validation AUC-ROC exceeded 0.90 for all intervention categories (Fig. 4A, B). For ventilation need (aggregating invasive and non-invasive ventilation), AUC-ROC at *T* = 19 was 0.962 on AmsterdamUMCdb compared to 0.992 on MIMIC-IV, with AUC-PR of 0.981 versus 0.991. For vasopressor need (aggregating norepinephrine, phenylephrine, epinephrine, dopamine), AUC-ROC was 0.937 versus 0.970, with AUC-PR of 0.838 versus 0.863. For inotrope need (dobutamine), AUC-ROC was 0.969 versus 0.988, with AUC-PR of 0.738 versus 0.947. Brier scores increased in external validation **(**Fig. 4C): from 0.031 (MIMIC-IV) to 0.044 (AmsterdamUMCdb) for ventilation need at *T* = 19, from 0.058 to 0.090 for vasopressor need, and from 0.002 to 0.010 for inotrope need. Reliability diagrams (Fig. 4D) show systematic overestimation of intervention probability in the external cohort.

Cold-start analysis was then replicated on AmsterdamUMCdb to assess onset prediction performance under external validation conditions (Table 6). Sample sizes were larger than MIMIC-IV: vasopressor onset *N* = 14,738, inotrope onset *N* = 1952, ventilation onset *N* = 5143. For vasopressor onset (*N* = 14,738, mean prevalence 70.66%), AUC-PR ranged from 0.607 (95% CI: 0.596–0.618) at *T* = 19 to 0.843 (0.835–0.850) at *T* = 24, with a mean AUC-PR of 0.730 (Table 6). For ventilation onset (*N* = 5143, prevalence 74.21%), AUC-PR ranged from 0.608 (0.590–0.627) to 0.892 (0.881–0.903), with a mean of 0.755. For inotrope onset (*N* = 1952, prevalence 77.75%), AUC-PR ranged from 0.634 (0.606–0.661) to 0.917 (0.899–0.933), with mean of 0.786. AUC-PR increased across the prediction horizon: ΔAUC-PR + 0.236 for vasopressor, +0.284 for ventilation, +0.283 for inotrope (*T* = 19 to *T* = 24). Confidence intervals were narrower than MIMIC-IV cold-start results (Table 5) due to larger sample sizes.

**Table 6:** Cold-start onset prediction performance on AmsterdamUMCdb external validation

Intervention	*N*	*T* = 19	*T* = 20	*T* = 21	*T* = 22	*T* = 23	*T* = 24	AUC-PR Mean
Vasopressor	14738	0.6069 (0.5959–0.6182)	0.6615 (0.6504–	0.7107 (0.7004–0.7207)	0.7583 (0.7484–0.7684)	0.8000 (0.7907–0.8088)	0.8427 (0.8350–0.8503)	**0.7300**
Inotropic	1952	0.6335 (0.6058–0.6607)	0.7176 (0.6896–0.7432)	0.7639 (0.7373–0.7889)	0.8159 (0.7917–0.8393)	0.8711 (0.8501–0.8916)	0.9166 (0.8990–0.9332)	**0.7864**
Ventilation	5143	0.6076 (0.5900–0.6270)	0.6695 (0.6511–0.6874)	0.7330 (0.7167–0.7499)	0.7880 (0.7726–0.8034)	0.8413 (0.8285–0.8546)	0.8918 (0.8808–0.9027)	**0.7552**

Intervention categories aggregate multiple agents: vasopressor (norepinephrine, phenylephrine, epinephrine, dopamine), inotropic (dobutamine, dopamine), ventilation (invasive and non-invasive). AUC-PR values with 95% confidence intervals from 1000 bootstrap iterations.

### GTM-based risk stratification

**Fig. 5:**
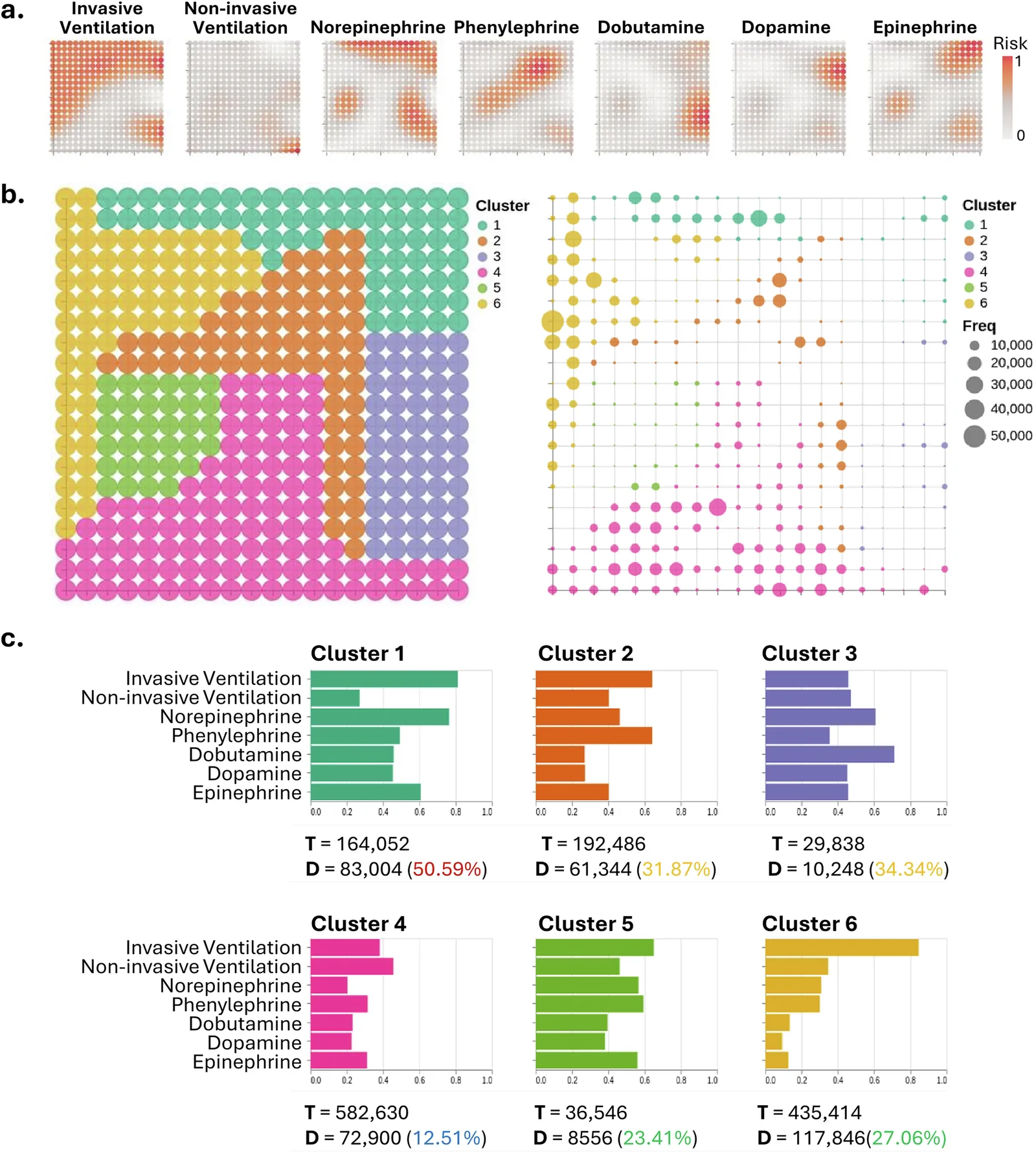
GTM-based risk stratification. **a** Reference vectors showing intervention probability distributions in the two-dimensional latent space. Colour intensity ranges from light grey (low probability) to dark red (high probability). **b** Membership map depicting the six macro-clusters and the number of hourly timesteps assigned to each micro-cluster. **c** Bar charts depicting the mean predicted intervention probabilities for each cluster alongside the mortality-associated trajectory percentage. Abbreviations: T number of hourly predictions, D associated deaths.

Using hourly intervention predictions from the temporal decoder, the GTM-based probabilistic interpretability module produced a two-dimensional latent space that revealed clinically distinct risk clusters (Fig. 5). The GTM configuration used a 20 × 20 latent grid (*L* = 400 nodes), *M* = 5 radial basis functions, RBF width *s* = 1, and regularisation parameter *λ* = 0.1, selected via grid search to maximise data log-likelihood while maintaining interpretable cluster structure.

Reference vector analysis showed intervention-specific regions in the latent space (Fig. 5a). Higher probability regions were observed for invasive ventilation in the upper-left quadrant, norepinephrine in the lower-right quadrant, and dobutamine overlapping with norepinephrine in the lower-right. Dobutamine-norepinephrine co-localisation and epinephrine-dopamine clustering in the upper-right were observed. Six macro-clusters were identified through agglomerative hierarchical clustering with Ward’s criterion applied to the 400 GTM reference vectors (Fig. 5b). The six-cluster solution was selected based on independent clinical review by two critical care physicians who identified phenotypes corresponding to established clinical categories. Quantitative assessment confirmed this choice: silhouette analysis across *K* = 2 to *K* = 8 revealed a plateau (scores 0.265–0.310), with *K* = 6 achieving a silhouette score of 0.308, indicating no single solution was statistically dominant and supporting clinical interpretability as the selection criterion. Stability assessment across five random initialisations demonstrated perfect agreement (Adjusted Rand Index = 1.000), confirming that the six-cluster solution is fully robust to initialisation variability (Supplementary Fig. 2).

Cluster 1 was characterised by mean predicted probabilities of 0.80 for invasive ventilation, 0.79 for norepinephrine, 0.51 for phenylephrine, and 0.62 for epinephrine. Cluster 2 showed 31.87% mortality-associated trajectories with the highest phenylephrine probability (0.64) across all clusters. Cluster 3 showed 34.34% mortality-associated trajectories with the highest dobutamine probability (0.75) across all clusters. Cluster 4 showed 12.51% mortality-associated trajectories with mean predicted probabilities below 0.35 for invasive ventilation and below 0.30 for all vasopressors. Cluster 6 showed 27.06% mortality-associated trajectories with the highest invasive ventilation probability (0.84) but lowest vasopressor requirements (norepinephrine 0.25, phenylephrine 0.28). Mortality outcomes were overlaid post hoc and did not influence GTM fitting or clustering.

**Fig. 6:**
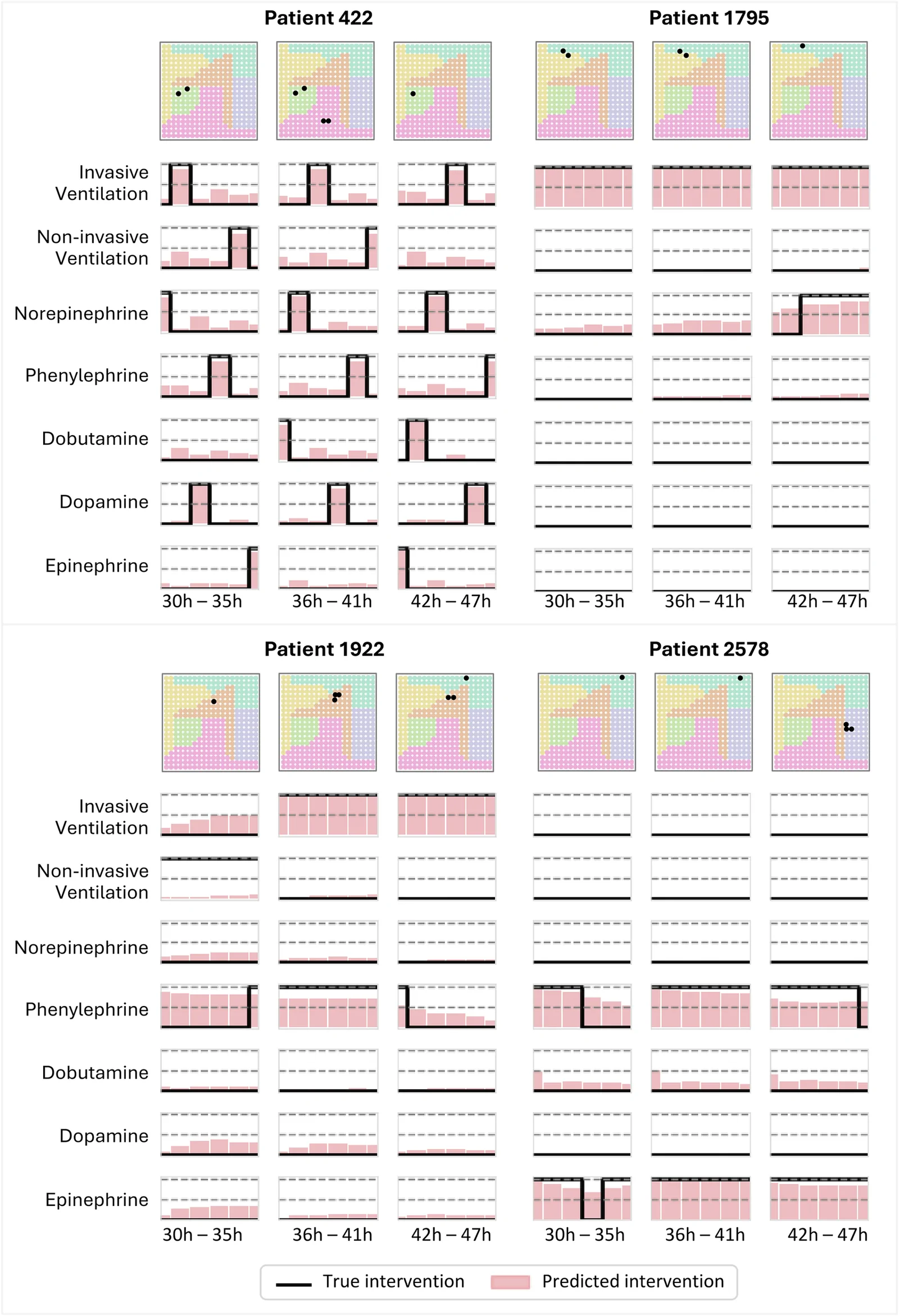
Individual patient trajectory analysis for four representative patients. For each patient, the top row shows GTM cluster assignments across three consecutive time intervals. Subsequent rows show bar graphs comparing predicted intervention probabilities (pink bars) against actual interventions administered (black lines). X-axes represent hours; y-axes represent probability (0-1). Patient demographics and vitals are provided in Table 7.

To illustrate how these macro-clusters relate to individual clinical courses, we examined four representative patients from different clusters and compared predicted intervention probabilities with the observed interventions administered over time (Fig. 6 and Table 7). For each patient, predicted intervention probabilities (pink bars) are compared against actual interventions administered (black lines) across three consecutive time intervals, with GTM cluster assignments shown in the top row.

**Table 7:** Demographics, ethnicity, and vital signs during the 24-h observation window for the four representative patients shown in Fig. 6

	Patient 422	Patient 1795	Patient 1922	Patient 2578
Age	89	89	60	85
Sex	Female	Male	Male	Female
Weight	68.66	62.9	87.4	76.9
Height	152.2	177.9	170.0	162.8
Ethnicity	White	White	Black	White
Temperature	36.1 [35.6, 36.8]	36.7 [36.4, 37.1]	36.1 [35.3, 36.9]	36.9 [36.6, 37.1]
Capillary refill	No	No	No	No
Diastolic BP	54.8 [39.0, 79.0]	52.0 [40.0, 65.0]	48.5 [43.0, 53.0]	50.8 [36.0, 96.0]
FiO2	50.4 [50.0, 55.0]	41.6 [40.0, 50.0]	75.0 [50.0, 100.0]	62.5 [50.0, 100.0]
GCS Total	13 [12,15]	9 [8, 14]	14 [11, 15]	14 [14, 15]
Glucose	173.0 [137.0, 186.0]	127.6 [65.0, 174.0]	143.7 [123.0, 196.0]	154.1 [121.0, 211.0]
Haemoglobin	9.8 [8.6, 10.0]	8.7 [8.1, 9.6]	9.9 [9.2, 10.1]	9.7 [8.9, 10.4]
Heart rate	92.61 [80.0, 122.0]	71.7 [62.0, 79.0]	64.8 [52.0, 77.0]	78.0 [64.0, 99.0]
Magnesium	2.3 [2.3, 2.3]	2.1 [1.8, 2.6]	2.7 [2.5, 2.9]	2.0 [1.8, 2.7]
Mean BP	63.0 [49, 83.3]	72.5 [58.0, 92.0]	67.6 [60.0, 72.3]	63.87 [46.0, 103.0]
Blood pH level	7.4 [7.4, 7.4]	7.4 [7.4, 7.4]	7.4 [7.4, 7.5]	7.4 [7.4, 7.4]
Platelet count	176.8 [141.0, 180.0]	177.8 [158.0, 198.0]	165.6 [143.0, 249.0]	114.3 [77.0, 183.0]
PT	29.8 [29.5, 33.6]	18.5 [17.0, 19.0]	11.4 [11.4, 11.4]	16.8 [15.3, 20.8]
Respiratory rate	22.0 [18.0, 29.0]	22.0 [17.0, 29.0]	19.9 [12.5, 28.0]	18.9 [10.0, 28.0]
SO2	94.2 [87.0, 97.0]	99.6 [95.0, 100.0]	94.0 [86.5, 97.5]	97.9 [92.0, 100.0]
Systolic BP	98.3 [80.0, 125.5]	111.6 [81.0, 147.0]	127.5 [105.5, 138.5]	106.7 [87.0, 130.0]
Anion Gap	15.7 [15.0, 18.0]	12.6 [10.0, 14.0]	12.5 [7.0, 17.0]	13.7 [10.0, 16.0]
Lactate	1.4 [1.3, 1.4]	1.0 [0.9, 2.0]	1.51 [0.7, 3.8]	3.4 [0.9, 5.2]
Phosphate	3.6 [3.5, 4.1]	4.6 [4.1, 5.7]	5.0 [3.6, 5.9]	3.5 [2.8, 4.9]
Creatinine	2.1 [2.0, 2.4]	1.6 [1.2, 1.9]	1.7 [1.2, 2.4]	0.9 [0.6, 1.0]

Continuous vital signs are reported as mean [min, max].

*BP* blood pressure, *FiO*_2_ fraction of inspired oxygen, *GCS* Glasgow Coma Scale, *PT* prothrombin time, *SO*_2_ oxygen saturation.

Patient 422 (89-year-old female) demonstrated accurate ventilation need prediction. FIRST-ICU captured ventilation requirements at hourly resolution, with the patient transitioning between GTM Clusters 5 and 4 before stabilising in Cluster 5 (Fig. 6). The temporal alignment between predicted needs, received interventions, and cluster transitions illustrates the interpretability enabled by probabilistic trajectory modelling.

Patient 1795 (89-year-old male) showed accurate vasopressor escalation prediction. The model predicted gradually increasing norepinephrine probability during the first two time intervals, with probabilities rising significantly in the third interval, aligning with actual norepinephrine administration at hour 44. GTM clustering tracked this clinical trajectory, showing movement from Cluster 6 to Cluster 1 as intervention probability increased (Fig. 6).

Patient 1922 (60-year-old male) demonstrated prediction of both intervention initiation and withdrawal. During the 30h–35 h interval, the model predicted a gradual increase in invasive ventilation risk reaching ~50% probability at 33 h, with similar patterns for phenylephrine. The model predicted higher phenylephrine probability starting at hour 30, while actual administration occurred at hour 35. In the final interval, predicted probabilities dropped significantly, and the true intervention was withdrawn at hour 43, consistent with model predictions. The patient alternated between Clusters 1 and 2, reflecting dynamic changes in clinical status (Fig. 6).

Patient 2578 (85-year-old female) illustrated the prediction of intervention discontinuation. For phenylephrine, the model predicted a gradual probability decline during the first interval, consistent with the intervention being stopped at hour 33. Regarding epinephrine, the model showed a probability drop at hour 33 with a subsequent increase; the true application mirrored this with withdrawal at hour 33 and reapplication at hour 34. The patient remained in Cluster 1 during the first two intervals, shifting to Cluster 3 as phenylephrine probability declined (Fig. 6).

## Discussion

This study presents FIRST-ICU, a unified deep learning framework that advances multi-intervention prediction in critical care through four key innovations: (1) joint modelling of intervention co-occurrence patterns via the IIAM, (2) validation of physiological learning through rigorous cold-start analysis, (3) establishment of cross-continental generalisability without retraining, and (4) integration of probabilistic clustering for clinically interpretable risk stratification. These contributions collectively address the fundamental limitation of existing approaches, namely, treating ICU interventions as independent events, while providing the interpretability essential for clinical translation.

The Temporal Decoder achieved exceptional discrimination with AUC-ROC exceeding 0.99 across all seven interventions and Brier scores consistently below 0.035 (Table 4), representing a substantial advance over single-task approaches while maintaining the calibration essential for clinical deployment^44,45^. For the Discrete Decoder, FIRST-ICU outperformed established baselines, including Suresh et al.^29^ (LSTM: 0.484, CNN: 0.480) and Xu et al.^22^ (GCNN: 0.500) on invasive ventilation prediction, achieving Macro AUC-PR of 0.514 (Table 3). Performance advantages were most pronounced for vasopressor interventions where co-administration patterns are clinically ubiquitous: norepinephrine improved from 0.354 (CNN) to 0.453 (+28.0%), while phenylephrine improved from 0.311 to 0.413 (+32.8%). These improvements arise from the multi-task formulation that enables shared learning across related prediction targets, consistent with theoretical foundations establishing that multi-task learning improves generalisation through implicit regularisation and inductive transfer^35,46^.

Importantly, FIRST-ICU achieved the highest Macro Average Precision for six of seven interventions and the highest Macro F1 for six of seven interventions, with non-overlapping confidence intervals over all baselines for invasive ventilation, norepinephrine, and phenylephrine. This consistency across multiple evaluation metrics strengthens confidence that performance gains reflect genuine improvements in clinical prediction rather than metric-specific optimisation artefacts.

Mechanistically, the ablation study provides evidence that the IIAM is a key contributor to performance across interventions and prediction horizons (Fig. 3). Removing the complete IIAM resulted in consistent performance degradation across all interventions and prediction horizons. The intervention interaction layer, implemented through 4-head self-attention, contributed most substantially to performance improvements. This component enables interventions to attend to each other, capturing co-administration patterns that single-task models cannot represent. Most vasopressor predictions showed the largest gains (phenylephrine ΔAUC-PR + 0.062, norepinephrine +0.049, dopamine +0.032), while ventilation interventions showed smaller but consistent improvements (invasive ventilation +0.007, non-invasive ventilation +0.034). It is important to note that the IIAM captures learned statistical associations between interventions rather than causal relationships. Establishing that one intervention causally influences the need for another would require interventional or counterfactual study designs, which are beyond the scope of this observational work. However, the alignment between the IIAM’s empirical contributions and established clinical co-administration practices provides evidence that the module captures patterns consistent with genuine treatment relationships.

The pattern of larger IIAM contributions for vasopressors compared to ventilation interventions admits a compelling clinical interpretation. Vasopressor co-administration is clinically common in refractory shock states: the VASST trial^47^ demonstrated that vasopressin co-administration with norepinephrine reduces catecholamine requirements in septic shock, while clinical practice guidelines recommend escalation to phenylephrine or epinephrine when norepinephrine monotherapy fails to achieve target mean arterial pressure^48^. By allowing interventions to explicitly attend to each other through self-attention, FIRST-ICU captures these statistical co-occurrence patterns directly from data without requiring manual feature engineering or domain-specific graph construction. The model discovers, for instance, that elevated norepinephrine probability increases phenylephrine probability, capturing the clinical pattern of vasopressor escalation in catecholamine-refractory shock.

Expert consensus documents that mechanical ventilation frequently necessitates vasopressor support due to cardiopulmonary interactions, including positive pressure-induced preload reduction and sedation-related vasodilation^49^. However, ventilation decisions are often more deterministic once respiratory failure criteria are met (PaO_2_/FiO_2_ ratio thresholds, work of breathing assessment), whereas vasopressor titration involves more dynamic multi-drug interactions. This difference may explain why the intervention interaction layer provides greater marginal benefit for vasopressor predictions.

The temporal attention mechanism addresses a distinct challenge: identifying which timepoints within the 12-h observation window are most informative for downstream prediction. In ICU settings, early warning signs often precede intervention requirements by several hours^28,50^. A declining oxygen saturation trajectory may predict ventilation need before overt respiratory failure, while subtle lactate elevation may herald vasopressor requirement before frank hypotension. The temporal attention mechanism contributed most significantly for interventions with complex temporal dynamics, such as ventilation weaning patterns where the timing of physiological readiness signals is critical. Without temporal attention, the model treats all timesteps with equal weight, potentially diluting critical signals from physiologically deteriorating periods. The shared encoder with layer normalisation^51^ provides feature transformation and training stability, ensuring consistent representations across the seven intervention prediction tasks while preventing gradient instabilities common in deep architectures processing heterogeneous clinical data.

Performance was not uniform across interventions, and the lower-prevalence targets, particularly dobutamine and dopamine, warrant careful interpretation. For dobutamine, LSTM achieved the highest Macro AUC-PR (0.467) compared to 0.443 for FIRST-ICU. For dopamine, GCNN showed the highest Macro AUC-PR (0.488) compared to 0.418 for FIRST-ICU, though FIRST-ICU achieved the highest Macro AP (0.427) and Macro F1 (0.460) for this intervention. These findings warrant a mechanistic explanation rather than dismissal as random variation.

Several factors likely contribute to differential performance on rare interventions. Importantly, the differential performance appears driven by pharmacological similarity rather than prevalence alone. Epinephrine, dobutamine, and dopamine have comparable hourly prevalence ( ~ 1.1%, ~0.9%, and ~0.7%, respectively; Table 4), yet FIRST-ICU achieved the highest performance across all three metrics for epinephrine (Table 3). This could be attributed to epinephrine being pharmacologically a vasopressor that shares co-administration dynamics with norepinephrine and phenylephrine, providing the intervention interaction layer with a meaningful cross-intervention signal.

In contrast, dobutamine and dopamine occupy distinct pharmacological niches where the intervention interaction layer may have limited signal to exploit. Dobutamine is the sole inotrope in our framework, used primarily for cardiogenic shock with reduced cardiac output^39,52^, a fundamentally different pathophysiology from the distributive shock states that drive norepinephrine and phenylephrine use. Its predictions likely depend more heavily on echocardiographic findings and cardiac output measurements than on other vasopressor trajectories, potentially limiting the benefit of cross-intervention attention. Dopamine’s dual inotropic and vasopressor properties at different dose ranges^38^ create heterogeneous use patterns that may not align cleanly with the co-administration dynamics learned from norepinephrine-phenylephrine interactions. Furthermore, the decline in dopamine use over recent decades^53,54^ may introduce temporal confounding in the MIMIC-IV database, which spans 2008-2019, as earlier admissions may reflect historical prescribing patterns while recent admissions favour norepinephrine as first-line therapy^55^. This temporal heterogeneity creates a non-stationary prediction target that could disadvantage models attempting to learn stable patterns across the full database.

Beyond pharmacological distinctions, extreme class imbalance further constrains multi-task learning benefits. Dobutamine onset occurred in only 0.1% of prediction windows, and dopamine onset in 0.2% (Table 2). Caruana’s foundational work on multi-task learning^35^ established that related tasks improve through shared representations, but tasks with fundamentally different statistical properties may experience negative transfer. When the decision boundary for rare interventions differs substantially from common interventions, forced parameter sharing can degrade performance compared to task-specific models^56,57^.

The metric-dependent performance pattern for dopamine (GCNN highest AUC-PR but FIRST-ICU highest AP and F1) suggests different models may optimise different aspects of the precision-recall trade-off. AUC-PR aggregates performance across all classification thresholds, while F1 reflects performance at a specific threshold optimised for that metric. For rare events, models may achieve high AUC-PR through well-calibrated probability estimates even when threshold-based classification performance (F1) is suboptimal, and vice versa. This divergence is common in highly imbalanced settings and does not necessarily indicate one model is categorically superior^58^.

These findings suggest that multi-task learning provides the greatest benefit when prediction targets share underlying pathophysiology (respiratory-circulatory interactions, vasopressor co-escalation) but may be less advantageous for pharmacologically distinct interventions with minimal co-administration patterns. Future work could explore hierarchical multi-task architectures that group related interventions (vasopressors, inotropes, ventilation) while allowing task-specific parameters for interventions with unique decision dynamics, potentially combining the benefits of shared learning with the flexibility to accommodate heterogeneous targets^59,60^.

An important methodological strength of this study is the cold-start analysis, which helps validate that FIRST-ICU is learning physiological precursors of intervention rather than merely extrapolating prior treatment history^61,62^. When current intervention status is included as a model input, predictions may simply extrapolate existing treatments rather than learn genuine physiological precursors, a form of information leakage that inflates apparent performance while providing minimal clinical utility. By evaluating performance specifically on cases where no intervention was present during the 12-hour observation window but at least one intervention occurred during the prediction window, we demonstrate that FIRST-ICU captures authentic early warning signals.

The maintained AUC-PR for cold-start cases, ranging from 0.667 for phenylephrine to 0.868 for invasive ventilation on MIMIC-IV (Table 5), indicates robust predictive capability from physiological signals alone. The consistent pattern of improving AUC-PR across the prediction horizon (invasive ventilation ΔAUC-PR + 0.225 from *T* = 19 to *T* = 24, norepinephrine +0.183, phenylephrine +0.201) indicates that physiological deterioration becomes increasingly apparent in hours immediately preceding intervention. This temporal gradient provides clinical validation that the model captures meaningful early warning signals rather than noise.

For vasopressor onset, the model likely learns patterns including early lactate elevation (a marker of tissue hypoperfusion), heart rate variability reduction (reflecting autonomic dysfunction), widening pulse pressure (indicating vasodilatory states), and subtle mean arterial pressure decline preceding overt hypotension. For ventilation, declining SpO_2_ trajectories, increasing respiratory rate, deteriorating work of breathing reflected in heart rate-respiratory rate coupling, and worsening PaO_2_/FiO_2_ ratios provide physiological signals predictive of intervention need.

This analysis has direct implications for clinical deployment. The highest-value predictions are those anticipating intervention needs before any treatment has begun, providing clinicians with early warning and preparation time that can materially affect patient outcomes. The 6-hour gap between observation and prediction windows already provides temporal separation, but the cold-start analysis confirms that even without any intervention signal in the observation window, the model successfully identifies patients who will require intervention initiation. Crucially, this finding was replicated on external data from AmsterdamUMCdb, where larger sample sizes (vasopressor N = 14,738, ventilation *N* = 5143) provided tighter confidence intervals and statistical power to confirm the robustness of physiological learning across healthcare systems (Table 6).

External validation on AmsterdamUMCdb (without retraining or recalibration) provides a stringent assessment of cross-continental generalisability, a critical requirement increasingly emphasised in clinical AI research^63,64^. The consistent AUC-ROC performance above 0.90 for all aggregated physiological need categories (Fig. 4A, B) demonstrates that learned patterns transfer across healthcare systems despite documented differences in treatment protocols between North America and Europe. This abstraction to physiological needs rather than specific drug choices was methodologically essential: phenylephrine prevalence differs by two orders of magnitude between cohorts (37.37% MIMIC-IV vs. 0.23% AmsterdamUMCdb), reflecting regional prescribing preferences rather than underlying patient physiology. Similarly, dopamine use is substantially higher in European practice (27.49% vs. 3.66%), while norepinephrine predominates in both settings as the first-line vasopressor.

Calibration showed degradation in external validation (Fig. 4C, D). Brier scores increased from 0.031-0.058 on MIMIC-IV to 0.044-0.090 on AmsterdamUMCdb, reflecting the well-documented phenomenon of calibration drift across clinical prediction models^36,65^. This degradation occurs because predicted probabilities, while maintaining rank-ordering (discrimination), may not correspond to true event frequencies in populations with different baseline intervention rates. The reliability diagrams (Fig. 4D) reveal systematic underestimation of intervention probability in the external cohort, consistent with substantially higher intervention prevalence in AmsterdamUMCdb (77.2% invasive ventilation vs. 63.8% MIMIC-IV).

These findings have important implications for multi-site deployment. While FIRST-ICU maintains strong discrimination across settings, the essential capability for risk stratification and identifying high-risk patients, local recalibration would be necessary before using predicted probabilities for threshold-based clinical decisions^63,65^. Methods such as Platt scaling, isotonic regression, or temperature scaling applied to local validation data could restore calibration without requiring full model retraining, an approach increasingly advocated for efficient clinical AI adaptation^64^. The preserved discrimination indicates that underlying learned representations generalise effectively, supporting the feasibility of calibration-only adaptation for new deployment sites.

The GTM-based post-prediction layer adds clinical interpretability by translating complex multi-intervention probability trajectories into phenotypes that may support risk stratification and decision-making. Unlike deterministic dimensionality reduction methods such as t-SNE or UMAP, GTM provides a principled probabilistic framework with explicit likelihood-based training via the expectation-maximisation algorithm^66^, enabling uncertainty quantification and direct out-of-sample projection of new patient trajectories onto the trained manifold, capabilities essential for real-time clinical deployment. The six macro-clusters identified (Fig. 5) represent distinct intervention phenotypes with differential clinical implications and mortality associations, providing clinicians with an intuitive framework for risk communication and treatment planning.

These six macro-clusters can be interpreted as distinct clinical phenotypes with characteristic intervention probability profiles and outcomes (Fig. 5C). Understanding these phenotypes may enable clinicians to contextualise individual patient trajectories within population-level patterns, though prospective validation would be required before implementation in clinical workflows.

Cluster 1 corresponded to a phenotype of combined respiratory-circulatory failure with multi-vasopressor support and the highest mortality. This phenotype represents the most critically ill patients, likely including those with septic shock complicated by acute respiratory distress syndrome (ARDS), or cardiogenic shock with pulmonary oedema requiring aggressive multi-system support^49,67^. The combination of high invasive ventilation probability with elevated norepinephrine and epinephrine probabilities suggests severe cardiopulmonary compromise with catecholamine-refractory shock requiring multi-agent vasopressor escalation. *Clinical Considerations*: Patients mapping to Cluster 1 may benefit from early intensivist review, consideration of advanced haemodynamic monitoring, and evaluation for escalation to advanced therapies (such as prone positioning, neuromuscular blockade, or extracorporeal support where appropriate criteria are met). Given the high mortality association (>50%), early goals-of-care discussions may be warranted in accordance with institutional practices.

Cluster 2 corresponded to respiratory failure with moderate circulatory support, characterised by a high phenylephrine pattern. This phenotype likely represents patients with respiratory failure requiring ventilatory support alongside vasopressor-responsive hypotension. The high phenylephrine probability may reflect perioperative or post-procedural patients in settings where phenylephrine is preferred for its pure alpha-agonist properties^68^, though prescribing patterns vary substantially between institutions and regions. The lower norepinephrine and inotrope probabilities suggest intact cardiac function with isolated vasoplegia. *Clinical Considerations*: Assessment for reversible causes of hypotension (such as residual anaesthetic effects, hypovolaemia, or neuraxial blockade), optimisation of volume status, and focus on ventilator weaning protocols may be appropriate. Monitoring for progression toward higher-acuity phenotypes (Cluster 1) could inform escalation decisions.

Cluster 3 corresponded to cardiogenic or inotrope-dependent shock, characterised by a high dobutamine pattern. This phenotype represents patients likely experiencing cardiogenic shock or septic cardiomyopathy requiring inotropic support for reduced cardiac output^52,67^. The high dobutamine probability combined with moderate norepinephrine suggests cardiac pump failure with secondary vasodilatory components. The relatively balanced invasive/non-invasive ventilation probabilities may reflect patients managed with non-invasive support or those with cardiac rather than primary pulmonary pathology. *Clinical Considerations*: Cardiology involvement, echocardiographic assessment if not recently performed, and evaluation for advanced cardiac support options may be appropriate for patients in this cluster. Monitoring for arrhythmias associated with inotropic therapy is typically indicated in such patients.

Cluster 4 represented a stable, low-acuity phenotype with minimal intervention requirements and the lowest mortality. This phenotype represents physiologically stable patients with minimal predicted intervention needs, potentially including those admitted for post-procedural monitoring, patients recovering from acute illness with improving trajectories, or lower-acuity admissions responding well to initial treatment. The low mortality association (12.5%) suggests favourable prognoses. *Clinical Considerations*: These patients may be appropriate candidates for step-down unit transfer evaluation or accelerated discharge planning, where clinically appropriate. However, continued trajectory monitoring remains important to identify unexpected deterioration (cluster transition).

Cluster 5 represented a mixed respiratory-circulatory support phenotype with intermediate acuity. This phenotype represents patients with multi-system involvement requiring balanced respiratory and circulatory support. The relatively even distribution across intervention types suggests patients in transitional states who may be improving from higher acuity (de-escalating from Cluster 1) or at risk of deteriorating from lower acuity (escalating toward Cluster 1). The intermediate mortality (23.4%) reflects this heterogeneous composition. Clinical *Considerations*: Frequent reassessment of trajectory direction, clear communication during clinical handoffs regarding the transitional nature of this phenotype, and defined escalation criteria may help identify patients requiring intensification of care versus those progressing toward recovery.

Cluster 6 represented a predominantly respiratory failure phenotype with comparatively favourable prognosis. This phenotype represents patients with primary respiratory pathology without significant circulatory compromise, potentially including pneumonia, acute exacerbations of chronic lung disease, post-operative respiratory failure, or neurological conditions affecting ventilation. The absence of significant vasopressor or inotrope requirement indicates intact cardiovascular reserve, which may explain the relatively favourable mortality (27.1%) compared to clusters with similar ventilation needs but added circulatory failure. *Clinical Considerations*: Focus on evidence-based ventilator management (lung-protective strategies), systematic assessment of extubation readiness, and monitoring for ventilator-associated complications aligns with standard respiratory failure management. The intact haemodynamics suggest potential for recovery with appropriate respiratory support.

From a clinical workflow perspective, the GTM cluster framework may provide a practical structure for ICU risk stratification that complements existing clinical assessment approaches. Rather than replacing clinical judgement, cluster-aware interpretation of predicted intervention trajectories may support proactive preparation alongside standard physiological monitoring. Table 8 presents an illustrative framework for how cluster assignments might inform clinical attention.

**Table 8:** Illustrative framework for cluster-informed clinical considerations

Cluster	Acuity level	Monitoringconsiderations	Potential proactive measures
1 (Multi-organ failure, 50.6% mortality)	Highest	Enhanced haemodynamic monitoring; consider cardiac output assessment	Early intensivist review; evaluate for advanced therapies; goals-of-care discussion
2 (Respiratory + vasopressor, 31.9% mortality)	Moderate-High	Ventilator weaning readiness; haemodynamic trends	Weaning protocols; assess for reversible hypotension causes
3 (Cardiogenic, 34.3% mortality)	High	Cardiac function monitoring; arrhythmia surveillance	Cardiology involvement; evaluate cardiac support options
4 (Stable, 12.5% mortality)	Low	Standard monitoring appropriate	Step-down/discharge evaluation; redirect resources as appropriate
5 (Transitional, 23.4% mortality)	Moderate	Trajectory monitoring; trend assessment	Define escalation/de-escalation criteria; clear handoff communication
6 (Respiratory only, 27.1% mortality)	Moderate	Ventilator parameters; extubation readiness	Lung-protective ventilation; systematic weaning assessment

These suggestions are hypothesis-generating and would require prospective validation before clinical implementation. Specific staffing models and monitoring frequencies should be determined by institutional policies and individual patient factors.

From a deployment perspective, FIRST-ICU addresses several practical requirements for ICU decision support that have historically limited clinical AI adoption. The 6-hour prediction horizon aligns with typical nursing shift durations and ICU rounding schedules, enabling anticipatory resource allocation and specialist consultation within operationally meaningful timeframes. The hourly probability outputs from the Temporal Decoder provide granularity beyond binary alerts: knowing that a patient has 20% vasopressor probability at hour 2 but 60% at hour 5 enables graduated clinical responses (preparation without premature intervention) rather than all-or-nothing alarms that contribute to alert fatigue^69^.

The multi-intervention framework explicitly addresses the clinical reality that ICU patients commonly require multiple concurrent therapies with complex interdependencies. Rather than presenting clinicians with seven independent alerts from single-intervention models (an approach virtually guaranteed to produce alert fatigue), FIRST-ICU provides a unified view of intervention trajectories that respects treatment co-occurrence patterns. The GTM clustering layer further summarises this high-dimensional output into six interpretable phenotypes, reducing cognitive load while preserving clinical nuance. This hierarchical approach (detailed probabilities for those who want them, summarised phenotypes for rapid assessment) accommodates diverse clinician preferences and workflow contexts.

Several deployment considerations warrant attention. First, the 24-hour minimum stay requirement for cohort inclusion means FIRST-ICU cannot make predictions for patients in their first hours of ICU admission, precisely when early warning may be most valuable. Alternative approaches, such as emergency department-based early warning systems or admission risk scores, could complement FIRST-ICU for this initial period. Second, real-time deployment requires infrastructure for streaming EHR data extraction, standardised preprocessing, and model inference at scale, technical challenges extending beyond algorithmic development that necessitate health informatics expertise and institutional IT support^24,64^. Third, integration with clinical workflows demands careful human-factors consideration: alert presentation format, escalation pathways, and documentation integration must be addressed through implementation science approaches, including clinician co-design and iterative refinement^70^.

In the context of contemporary clinical time-series modelling, the architecture of FIRST-ICU offers a useful point of comparison with recent advances in clinical time series modelling. Transformer-based approaches^21,71^ have demonstrated strong performance for capturing long-range dependencies in sequential data, but the 12-hour observation window used here may not require the extended context lengths where transformers excel over recurrent architectures. The LSTM backbone provides computational efficiency and proven effectiveness for clinical sequences at this temporal scale^17,72^, while the IIAM adds targeted attention mechanisms specifically designed for intervention prediction without the quadratic complexity of full self-attention across all timesteps.

GNNs for clinical data have gained substantial attention for their ability to model inter-variable relationships^23,28,73^. FIRST-ICU’s GNN encoder learns a fully-connected graph over clinical variables through message passing, enabling the model to discover relationships such as blood pressure-vasopressor correlations and lactate-ventilation associations without requiring explicit graph structure specification or domain knowledge engineering. This data-driven approach to relationship learning provides flexibility across clinical contexts where optimal graph structures may not be known a priori.

The multi-task learning framework extends established benchmarks^74^ beyond standard parameter sharing. The intervention interaction layer in the IIAM enables task-specific representations to explicitly influence each other through cross-intervention attention, a mechanism that may explain performance advantages over baselines using simpler shared-encoder approaches without inter-task communication. Recent work on sequential subnetwork routing^75^ and modular multi-task learning has explored similar ideas of flexible, task-aware parameter sharing, representing promising directions for future architectural refinements.

Several limitations should be acknowledged when interpreting these findings. First, exclusion of admissions shorter than 24 h introduces selection bias toward patients with extended ICU stays, potentially limiting generalisability to rapid-turnover populations such as post-procedural monitoring or observation admissions. While this decision was methodologically necessary for constructing observation and prediction windows of adequate length, the clinical characteristics of excluded patients may differ systematically, as these are often either very stable (brief observation) or very unstable (early death) patients.

Secondly, class imbalance remains substantial despite focal loss and weighted cross-entropy. Transition states (onset 0.1–1.8%, wean 0.1–2.4%) occur far less frequently than stable states (stay on/off >95%), as quantified in Table 2. The rarest intervention-state combinations (dobutamine onset 0.1%, dopamine onset 0.2%) have limited sample sizes that constrain statistical power and may produce optimistic or unstable performance estimates reflected in wide bootstrap confidence intervals. As discussed earlier, this extreme rarity may also limit the benefits of multi-task learning for these specific targets.

Thirdly, temporal validation was not performed due to database constraints. Training and test splits were random rather than chronologically ordered, meaning potential performance degradation over time due to evolving clinical practices, changing patient populations, or documentation pattern shifts was not assessed. However, the external validation on AmsterdamUMCdb partially mitigates this concern, as it represents a different institution, country, time period, and prescribing practice, with maintained AUC-ROC above 0.90 without retraining. A chronological train/test split on MIMIC-IV (e.g. training on 2008–2015, testing on 2016–2019) would provide a more rigorous assessment of temporal stability and should be explored in future work. Calibration drift is a well-documented phenomenon in deployed clinical prediction models^36,65^, and prospective temporal validation would be essential before real-world implementation.

Fourth, the current framework predicts binary intervention occurrence rather than dosage or titration strategy. In ICU management, dose gradients and combined titration strategies are clinically important, and predicting only whether an intervention will occur does not capture this granularity. However, binary onset prediction serves a complementary purpose as the critical first decision point for resource allocation and clinical preparedness, with dose optimisation representing an important downstream extension.

Fifth, the hourly temporal averaging of clinical measurements within each time bin discards potentially informative signal from measurement frequency. A clinician ordering multiple blood draws within a single hour signals heightened concern about near-horizon deterioration, and this information is lost when measurements are averaged. Incorporating measurement frequency as an additional feature represents a promising direction for future work.

Sixth, the GTM phenotype clusters are generated from predicted intervention probability trajectories and do not control for confounding factors such as age, comorbidities, infection type, or ICU management practices, all of which influence both intervention patterns and mortality outcomes. Since mortality was overlaid post hoc, the observed mortality associations may partly reflect these confounders. The phenotypes therefore, provide risk stratification and patient characterisation rather than causal treatment guidance, and disentangling these effects would require propensity adjustment or causal inference frameworks.

Additionally, external validation was performed on a single European cohort. While AmsterdamUMCdb provides a valuable cross-continental assessment, generalisability to other healthcare systems, particularly resource-limited settings with different intervention thresholds, monitoring capabilities, and staffing models, remains uncertain. Multi-centre validation across diverse settings, including community hospitals, low-resource environments, and non-Western healthcare systems would strengthen confidence in model robustness.

Finally, GTM cluster interpretations, while clinically grounded and validated through mortality association, require prospective clinical validation. The proposed workflow modifications (Table 8) represent expert-informed recommendations rather than empirically validated care pathways. Cluster-based decision-making should be evaluated through implementation studies assessing both clinical outcomes and workflow feasibility before widespread adoption.

Several directions for future research follow directly from this work. The most immediate priority is prospective validation in real-world clinical settings, evaluating patient outcomes (mortality, length of stay, intervention appropriateness) rather than prediction accuracy alone. Such studies would establish clinical utility beyond algorithmic performance while incorporating clinician feedback on decision support utility and workflow integration feasibility.

The multi-task framework naturally accommodates expansion to additional intervention types, including renal replacement therapy, blood product transfusion, sedation management, and nutrition support, which would provide more comprehensive critical care decision support. Based on our findings regarding differential performance across pharmacologically distinct interventions, hierarchical multi-task architectures that group related interventions while allowing task-specific parameters may optimise performance across diverse targets^59,60^, though computational and data requirements would scale accordingly. Broader clinical adoption would also benefit from efficient recalibration procedures enabling adaptation to new deployment sites without full retraining. Transfer learning approaches, domain adaptation techniques, and lightweight recalibration methods, such as temperature scaling, merit investigation for portable deployment across heterogeneous healthcare systems.

Beyond structured clinical variables, incorporation of additional data modalities such as clinical notes, imaging findings, and continuous waveform data could enhance prediction performance, particularly for interventions where structured data provides incomplete signals. The GNN encoder architecture naturally accommodates heterogeneous inputs through node-level feature concatenation. Incorporating measurement frequency as a feature, rather than averaging multiple measurements within temporal bins, could capture the clinical signal embedded in monitoring intensity. A clinician ordering frequent measurements signals concern about deterioration, and this information could enhance early warning capabilities, particularly for the cold-start prediction scenario.

Encoder architecture comparison represents another important direction. While the GNN encoder provides relational inductive biases suited to clinical variable interactions, systematic comparison with alternative backbone architectures (CNNs, MLPs, Transformers) would quantify the specific contribution of graph-based encoding to multi-intervention prediction performance. More broadly, transformer-based and graph-based clinical architectures designed for general EHR outcome prediction represent approaches that have also demonstrated strong performance on mortality, phenotyping, and length-of-stay benchmarks. Adapting such architectures to the simultaneous multi-label intervention prediction at hourly resolution task, where inter-intervention dependencies, temporal dynamics, and class imbalance must be jointly addressed, could represent a promising direction for future work.

Looking further ahead, causal inference methods could extend FIRST-ICU beyond prediction toward treatment effect estimation, enabling not just forecasting of what interventions will occur but recommendations for what interventions should occur. This substantially more ambitious objective would require careful methodological development to address confounding and selection bias inherent in observational data, yet represents the ultimate goal of moving from predictive to prescriptive decision support in critical care.

In summary, FIRST-ICU represents a substantial advance in multi-intervention prediction for critical care, addressing fundamental limitations of existing single-task approaches through joint modelling of treatment co-occurrence patterns, validated physiological learning, cross-continental generalisability, and interpretable risk stratification. The integration of the IIAM captures clinically meaningful co-administration patterns, while cold-start analysis confirms that predictive signals arise from genuine physiological precursors rather than intervention history extrapolation. External validation demonstrates that learned representations transfer across healthcare systems when abstracted to physiological needs, supporting the potential for portable deployment.

The GTM-based clustering provides an interpretability layer that translates complex probability trajectories into six clinically interpretable phenotypes with differential mortality associations, enabling risk stratification that complements clinical assessment. By enabling clinicians to visualise not just the current patient state but also the predicted trajectory direction, FIRST-ICU supports proactive rather than reactive care, the fundamental promise of clinical decision support in intensive care. The combination of fine-grained temporal forecasting, validated cross-institutional performance, and clinically grounded phenotyping positions FIRST-ICU as a comprehensive framework for advancing ICU decision support toward personalised, anticipatory critical care.

## Methods

### Data source

The primary database used in this study was derived from the Medical Information Mart for Intensive Care IV (MIMIC-IV), a publicly available hospital database that contains detailed clinical data for over 257,000 unique patients and 524,000 hospital admissions. We used version 2.2, which includes high-resolution records of vital signs, laboratory measurements, medications, procedures, and intervention timelines for 50,764 patients and 73,181 ICU stays^9^. The longitudinal and time-stamped structure of MIMIC-IV enables the analysis of temporal relationships between evolving patient physiology and treatment decisions, making it *well-suited* for developing models that forecast multiple ICU interventions.

For external validation, we employed the AmsterdamUMCdb^10^, a large, de-identified critical care database from the Amsterdam University Medical Centres. This database contains detailed physiological measurements, interventions, and outcomes from a European ICU population. The same extraction and preprocessing pipeline developed for MIMIC-IV was applied to AmsterdamUMCdb to ensure methodological consistency and enable direct comparison of model performance across healthcare systems, thereby assessing the cross-continental generalisability of the learned representations.

### Data extraction and preprocessing

For this study, we extracted hourly sequences of patients’ vitals (e.g. heart rate, respiratory rate, and temperature), laboratory results (e.g. lactate and phosphate levels), drugs administered (e.g. dopamine, dobutamine), procedures carried out (e.g. invasive and non-invasive ventilation), and other clinical demographic information (e.g. age, sex, ethnicity). Patients younger than 18 years of age and those with multiple ICU stays were excluded from the analysis.

Admissions lasting less than 24 h were also excluded from the analysis. This exclusion criterion was implemented for several reasons: such admissions do not contain sufficient longitudinal information to construct the required temporal observation and prediction windows; they often represent brief monitoring episodes or rapid transfers rather than complete ICU trajectories; and including such cases would introduce truncated sequences that distort temporal pattern learning. This decision was further supported by expert clinical guidance, which indicated that short admissions are not physiologically comparable to standard ICU stays and would reduce the interpretability and clinical validity of temporal predictions.

The data for modelling was formatted into a three-dimensional structure representing admissions, hourly sequences, and features (i.e. vitals, lab tests, etc.). Vital and laboratory measurements were timestamped to the nearest hour, and multiple measurements within an hour were averaged. Static demographic variables were replicated across all timesteps. Numerical variables were standardised (i.e. mean-centred with a standard deviation of 1), and categorical variables were one-hot encoded.

Missing values were handled as follows: The last observation carried forward (LOCF) method was used to fill gaps in continuous time series. Admissions with specific vitals missing were imputed using Multiple Imputation by Chained Equations (MICE)^76^. Hourly periods with no recorded procedure or drug events were assumed to indicate that the patient did not receive interventions during that period. The extent of missingness varied substantially across variable types. Continuously monitored vital signs exhibited very low missingness: heart rate (0.1%), temperature (0.2%), blood pressure (0.1%), respiratory rate (0.2%), and oxygen saturation (0.4%). Laboratory values obtained through intermittent sampling showed moderate missingness: glucose (10%), haemoglobin (20%), platelet count (19%), and blood pH (17.54%). Certain variables had higher missingness due to clinical documentation practices: fraction of inspired oxygen (24.44%), capillary refill (26.95%), prothrombin time (24.90%), and weight (22.17%). Static demographic variables (age) were complete for all admissions. Missing values were handled through a two-stage approach: Last Observation Carried Forward (LOCF) was first applied to fill gaps in continuous time series, reflecting the clinical assumption that the most recent measurement remains the best estimate until a new measurement is obtained. The remaining missing values were then imputed using Multiple Imputation by Chained Equations (MICE), which generates plausible values based on the observed distribution of related variables. We acknowledge that this imputation strategy may introduce bias when missingness is not at random.

Temporal vital and laboratory variables, static demographic variables, and intervention variables (procedures and drug events as binary indicators) were concatenated into a single feature vector for each patient admission at every hourly timestep.

To support both categorical and hour-by-hour forecasting, FIRST-ICU produces forecasts using two alternative decoder heads operating on a shared encoded patient state: a Discrete Decoder that predicts coarse-grained intervention states over fixed windows, and a Temporal Decoder that predicts hour-by-hour intervention trajectories. The model operates on a 12-h observation window for encoding patient state, followed by a 6-h gap period and a subsequent prediction window (Fig. 1). The 6-h gap period serves a specific clinical purpose: it provides actionable lead time for clinicians to prepare for anticipated interventions. This separation allows time for resource preparation (e.g. central line placement for vasopressors, ventilator availability), specialist consultation, and anticipatory planning (e.g. staffing adjustments, medication preparation). This design choice was informed by clinical input and aligns with prior work in this domain^22,28,29,50^. Without this gap, predictions would concern the immediate future, where clinicians may already be aware of intervention needs or have insufficient time to prepare. The duration represents a trade-off between prediction accuracy and clinical utility, validated by the strong cold-start performance (Table 5), demonstrating that the model captures physiological warning signals well before intervention initiation. The duration of the prediction window varies by decoder variant: the Discrete Decoder uses a 4-h prediction window following Suresh et al.^29^, while the Temporal Decoder uses a 6-h prediction window to provide extended forecasting horizons for clinical deployment scenarios. Notably, although admissions shorter than 24 h were excluded during cohort selection, the 12-h observation window means the model can still process patients with stays as short as 24 h, as only 12 h of observation data plus the gap and prediction windows are required.

Two complementary target representations were defined to support the two decoder variants of FIRST-ICU, enabling both direct comparison with prior work and fine-grained temporal forecasting.

For the Discrete Decoder, intervention trajectories within the 4-h prediction window were mapped to four mutually exclusive categorical states following Suresh et al.^29^. ONSET was assigned when an intervention transitioned from off (0) to on (1) at any time within the window. WEAN was assigned when an intervention transitioned from on (1) to off (0). STAY ON was assigned when the intervention remained continuously on throughout the window, and STAY OFF when it remained continuously off. This formulation enables direct comparison with existing state-of-the-art methods that use categorical state prediction, providing a standardised and widely adopted evaluation framework. In prior work^22,29^, discrete state labels were preferred over hourly labels because one-hour switch events produce impulse-like targets that exacerbate class imbalance, with transition states occurring far less frequently than stable states, which can hinder learning and reduce model robustness. However, this discretisation collapses fine-grained temporal dynamics and obscures the precise timing of intervention changes, limiting its suitability for real-time clinical forecasting.

For the Temporal Decoder, the original hourly binary intervention indicators were retained for each hour in the 6-hour prediction window and for each intervention. Let $k\in \{1,\ldots ,K\}$ index interventions and $t\in \{1,\ldots ,T\}$ index hours within the prediction window, where T=6. The target variable is defined as

1 $${y}_{t,k}\in \{0,1\}$$

where

2 $${y}_{t,k}=\left\{\begin{array}{l}1,{\mathrm{if}}\,{\mathrm{intervention}}\,k\,{\mathrm{is}}\,{\mathrm{administered}}\,{\mathrm{at}}\,{\mathrm{hour}}\,t\\ 0,\,\mathrm{otherwise}\end{array}\right.$$

This formulation preserves the full temporal resolution of intervention trajectories, enabling the model to predict the precise timing and duration of therapy rather than collapsing all changes within a window into a single category. Unlike discrete state labels, which discard when transitions occur, the temporal formulation supports fine-grained forecasting of both onset timing and treatment persistence across multiple concurrent therapies. This granularity is particularly valuable for real-time ICU decision support, where knowing that a patient is likely to require vasopressors in two hours rather than five hours can materially affect clinical preparedness and resource allocation.

### Description of the proposed methodology

FIRST-ICU employs a Graph Neural Network Autoencoder (GNN-AE) architecture comprising three main components (Fig. 2). The first is a GNN-based encoder, FIRST-GraphEncoder, which transforms multivariate clinical time series into deterministic latent representations by learning inter-variable relationships through message passing. This encoder is adapted from a previously proposed neural time series graph encoder^77^, with modifications for ICU multi-intervention forecasting. The second component is the intervention interaction attention module (IIAM), which processes LSTM outputs through temporal attention, shared feature encoding, and intervention interaction layers. Finally, the prediction heads generate intervention forecasts from the IIAM outputs. The two decoder variants share identical architectures, including the IIAM, and differ only in their final prediction heads, enabling fair comparison between categorical and temporal prediction paradigms while maintaining architectural consistency.

The input to the encoder is a tensor ${\bf{X}}\in {{\mathbb{R}}}^{B\times T\times N\times F}$, where B is the batch size, T is the context length (hours), N is the number of clinical variable nodes, and F is the feature dimension per node. The model predicts intervention states for seven interventions: non-invasive ventilation, invasive ventilation, norepinephrine, phenylephrine, dopamine, epinephrine, and dobutamine. This multi-task formulation is designed to capture statistical co-occurrence patterns between interventions that would be missed by independent single-intervention models.

The first component is the FIRST-GraphEncoder, which transforms multivariate clinical time series into a latent representation by treating clinical variables as nodes in a fully-connected graph and learning their inter-relationships through message-passing operations. This design is motivated by the observation that ICU variables are not independent; for example, blood pressure changes may correlate with vasopressor administration, while respiratory parameters relate to ventilation status.

The choice of a GNN encoder is motivated by the relational inductive bias that graph-based architectures provide. By constructing each patient’s clinical data as a fully-connected graph with variables as nodes, the message-passing mechanism learns which variable-to-variable relationships are informative through trainable edge functions (Algorithm 1). This differs from alternative architectures in how inter-variable relationships are treated. CNNs impose fixed spatial or temporal adjacency, assuming nearby features in the input vector are related, which is arbitrary for clinical variables that have no natural ordering. Multi-layer perceptrons treat all features as independent inputs, lacking explicit relational structure. While attention-based models can learn pairwise relationships, they do so without the iterative neighbourhood aggregation that gives GNNs their capacity to capture higher-order relationships between variables. Our encoder employs two rounds of message passing, allowing information to propagate between nodes that are two hops apart, effectively capturing second-order physiological relationships (e.g. the indirect relationship between lactate and ventilation mediated through blood pressure). This relational inductive bias aligns with the theoretical framework established by Battaglia et al.^78^, who demonstrated that graph networks provide a principled approach for learning over structured relational data.

Given the input tensor ${\bf{X}}\in {{\mathbb{R}}}^{B\times T\times N\times F}$, the encoder first reshapes the data to $\left(B\cdot N\right)\times \left(T\cdot F\right)$, treating each variable’s time series as a node feature vector. The encoding proceeds through multiple message-passing layers with mathematically defined aggregation operations.

#### Graph structure

We construct a fully-connected graph (excluding self-loops) over N nodes representing clinical variables. Let E denote the set of directed edges, where $\left|E\right|=N\left(N-1\right)$. We define two binary relation matrices:

3 $${\bf{R}}\in \{0,1{\}}^{\left|E\right|\times N}\,(\mathrm{receiver\; matrix})$$

4 $${\bf{S}}\in \{0,1{\}}^{\left|E\right|\times N}\,(\mathrm{sender\; matrix})$$

where ${R}_{e,v}=1$ if edge e has node v as its receiver, and ${S}_{e,v}=1$ if edge e has node v as its sender. These matrices encode the graph topology and enable efficient batch computation of message passing.

#### Node-to-edge aggregation (node2edge)

This operation constructs edge representations by concatenating features from sender and receiver nodes. Given node representations ${\bf{h}}\in {{\mathbb{R}}}^{N\times H}$, the edge features are computed as:

5 $${\bf{e}}=\left[{\bf{R}}\cdot {\bf{h}};{\bf{S}}\cdot {\bf{h}}\right]\in {{\mathbb{R}}}^{\left|E\right|\times 2H}$$

where $\left[\cdot ;\cdot \right]$ denotes concatenation along the feature dimension, ${\bf{R}}\cdot {\bf{h}}$ extracts receiver node features for each edge, and ${\bf{S}}\cdot {\bf{h}}$ extracts sender node features. This produces edge representations that capture pairwise relationships between clinical variables.

#### Edge-to-node aggregation (edge2node)

This operation aggregates incoming edge messages to update node representations. Given edge features ${\bf{e}}\in {{\mathbb{R}}}^{\left|E\right|\times H}$, the updated node representations are computed as:

6 $${{\bf{h}}}^{{\boldsymbol{{\prime} }}}=\frac{1}{\left|E\right|}\cdot {{\bf{R}}}^{\top }\cdot {\bf{e}}\in {{\rm{{\mathbb{R}}}}}^{N\times H}$$

where ${{\bf{R}}}^{{\rm{\top }}}\cdot {\bf{e}}$ sums edge features for all edges incident to each receiver node, and the normalisation factor $1/\left|E\right|$ ensures scale invariance. This aggregation enables each node to incorporate information from all other nodes in the graph.

##### Algorithm 1

Message Passing Encoder Architecture

**Require:** Node features $X\in {{\mathbb{R}}}^{B\times T\times N\times F}$, graph $\left(V,|,E\right)$, hidden dimension H

**Ensure:** Latent node representation $\mu \in {{\mathbb{R}}}^{B\times N}$

1. Reshape $$X\to (B\cdot N)\times (T\cdot F)$$

2. Compute ${h}^{\left(0\right)}\leftarrow {\mathrm{MLP}}_{1}(\mathrm{reshape}(X))$*(ELU + dropout)*

3. $${e}^{\left(0\right)}\leftarrow \mathrm{node}2\mathrm{edge}({h}^{\left(0\right)})$$

4. ${e}^{\left(1\right)}\leftarrow {\mathrm{MLP}}_{2}({e}^{\left(0\right)})$, save as skip

5. $${h}^{\left(1\right)}\leftarrow \mathrm{edge}2\mathrm{node}({e}^{\left(1\right)})$$

6. $${h}^{\left(2\right)}\leftarrow {\mathrm{MLP}}_{3}({h}^{\left(1\right)})$$

7. $${e}^{\left(2\right)}\leftarrow [\mathrm{node}2\mathrm{edge}({h}^{\left(2\right)});{e}^{\left(1\right)}]$$

8. $${e}^{\left(3\right)}\leftarrow [{e}^{\left(2\right)};{e}^{\left(1\right)}]$$

9. ${e}^{\left(4\right)}\leftarrow {\mathrm{MLP}}_{4}({e}^{\left(3\right)})$*(ELU, no dropout)*

10. $${h}^{\mathrm{final}}\leftarrow \mathrm{edge}2\mathrm{node}({e}^{\left(4\right)})$$

11. $$\mu \leftarrow {\mathrm{Linear}}({h}^{\mathrm{final}})$$

All MLP layers use ELU activation functions and dropout regularisation, except for the final linear projection and the hidden transformation layer (no dropout). The encoder architecture employs two rounds of message passing, allowing information to propagate between nodes that are two hops apart in the graph, effectively capturing second-order relationships between clinical variables. The hidden dimension H is set to the encoder hidden dimension specified in the model parameters.

The encoded patient representation is then processed by a shared decoder architecture comprising a recurrent temporal backbone and the IIAM, which together generate intervention-specific representations for downstream prediction. Both decoder variants share this same decoder architecture and differ only in their final prediction heads. This design ensures that any performance differences between the Discrete and Temporal decoders arise from the output formulation rather than architectural confounders.

#### Decoder input representation

The decoder receives a latent representation ${\bf{z}}\in {{\mathbb{R}}}^{B\times N}$, where B is the batch size and *N* is the number of clinical variable nodes. This latent graph embedding summarises inter-variable dynamics over the 12-h observation window. To condition predictions on the most recent physiological context, **z** is concatenated with the observed features from the final 6 h of the observation window (hours 7–12). Let ${{\bf{X}}}_{{\rm{recent}}}\in {{\mathbb{R}}}^{B\times 6\times N}$ denote the observed input features over the final 6 h. The received latent representation is replicated across time to form ${{\bf{z}}}_{{\rm{expanded}}}\in {{\mathbb{R}}}^{B\times 6\times N}.$

The decoder input is then ${{\bf{X}}}_{{\rm{dec}}}=[{{\bf{X}}}_{{\rm{recent}}};{{\bf{z}}}_{{\rm{expanded}}}]\in {{\mathbb{R}}}^{B\times 6\times 2N}.$ This construction allows the decoder to jointly condition on recent temporal trends and the global latent structure learned by the graph encoder.

#### Recurrent temporal modelling

The decoder input ${{\bf{X}}}_{{dec}}$ is processed by a two-layer LSTM with hidden size 256. The LSTM serves as the temporal backbone, transforming the concatenated latent and observed features into a sequence of hidden states that encode temporal dynamics within the 6-h prediction window. The LSTM outputs a sequence of hidden representations

7 $${\bf{H}}=\{{h}_{1},{h}_{2},\ldots ,{h}_{6}\}\in {{\mathbb{R}}}^{B\times 6\times 256}$$

where each ${h}_{t}$ summarises the model’s internal state at the prediction hour *t* conditioned on both the learned latent graph representation and recent observed clinical measurements. These hidden states are then passed to the IIAM for temporal weighting and intervention-level interaction modelling.

### Intervention interaction attention module (IIAM)

The IIAM transforms the sequence of LSTM hidden states into intervention-specific representations that explicitly encode temporal relevance and cross-intervention dependencies. Given the LSTM outputs ${\bf{H}}=\{{h}_{1},{h}_{2},\ldots ,{h}_{6}\}\in {{\mathbb{R}}}^{B\times 6\times 256}$, the IIAM first applies a learnable temporal attention mechanism to weight timesteps according to their importance for downstream intervention prediction. For each timestep t, an attention energy is computed as

8 $${e}_{t}={{\bf{w}}}_{2}^{\top }\tanh ({{\bf{W}}}_{1}{h}_{t})$$

where ${{\bf{W}}}_{1}\in {{\mathbb{R}}}^{H/2\times H}$ and ${{\bf{w}}}_{2}\in {{\mathbb{R}}}^{H/2}$ are learnable parameters. These energies are normalised with a softmax to obtain attention weights

9 $${\alpha }_{t}=\frac{\exp \left({e}_{t}\right)}{{\sum }_{j=1}^{6}\exp ({e}_{j})}$$

and the sequence is summarised into a context vector

10 $${\bf{c}}=\mathop{\sum }\limits_{t=1}^{6}{\alpha }_{t}{h}_{t}$$

This operation allows the model to focus on the most informative portions of the recent clinical trajectory rather than treating all timesteps equally.

The attention-weighted context ${\bf{c}}$ is then passed through a shared feature encoder that produces a normalised latent representation used by all intervention heads. This shared encoder is defined as

11 $${{\bf{f}}}_{{\mathrm{shared}}}={\mathrm{Dropout}}({\mathrm{GELU}}({\mathrm{LayerNorm}}({{\bf{W}}}_{s}{\bf{c}}+{{\bf{b}}}_{s})))$$

where ${{\bf{W}}}_{s}\in {{\mathbb{R}}}^{H\times H}$ and ${{\bf{b}}}_{s}\in {{\mathbb{R}}}^{H}$ are learnable parameters. Layer normalisation stabilises optimisation across batches and time, while the nonlinearity and dropout encourage robust feature learning. This shared representation acts as a common clinical embedding from which all intervention predictions are derived, enforcing consistent use of temporal information across tasks.

To model interactions between interventions, the shared representation is expanded into an intervention-indexed tensor ${\bf{F}}\in {{\mathbb{R}}}^{B\times K\times H}$, where *K* is the number of interventions. Each slice ${{\bf{F}}}_{:,k,:}$ corresponds to intervention *k*and is processed using multi-head self-attention, allowing interventions to attend to one another. Using the standard scaled dot-product attention, queries, keys, and values are computed as linear projections of ${\bf{F}}$, and attention is applied as

12 $$\mathrm{Attention}({\bf{Q}},{\bf{K}},{\bf{V}})=\mathrm{softmax}\left(\frac{{\bf{Q}}{{\bf{K}}}^{\top }}{\sqrt{{d}_{k}}}\right){\bf{V}}$$

Multi-head attention is implemented by concatenating four such attention heads followed by a linear projection, and is combined with residual connections, layer normalisation, and a feed-forward network to produce the final intervention-specific representations ${{\bf{F}}}_{\mathrm{out}}\in {{\mathbb{R}}}^{B\times K\times H}$. This interaction layer enables the model to capture clinically meaningful co-administration patterns, such as the frequent joint use of multiple vasopressors or the transition between ventilation modalities.

Together, temporal attention, shared feature encoding, and intervention-level self-attention provide a unified mechanism that first identifies informative time points, then extracts a common clinical state, and finally models interventional dependencies, enabling coherent multi-intervention forecasting from complex ICU trajectories.

From these intervention-specific representations, two alternative prediction heads are used interchangeably depending on the task: a discrete decoder for categorical state prediction and a Temporal Decoder for hour-by-hour probability forecasting (only a single head is instantiated at a time). These heads map the intervention-specific representations ${{\bf{F}}}_{{\rm{out}}}[:,k,:]\in {{\mathbb{R}}}^{B\times H}$ to their respective output spaces for each intervention k.

#### Discrete decoder head

For each intervention k, the model estimates a categorical distribution over the four states:

13 $${{\bf{p}}}_{k}={\rm{softmax}}({f}_{k}({{\bf{F}}}_{{\rm{out}}}[:,k,:]))\in {[0,1]}^{B\times 4},\mathop{\sum }\limits_{c=1}^{4}{p}_{k,c}=1$$

where ${f}_{k}:{{\mathbb{R}}}^{H}\to {{\mathbb{R}}}^{4}$ is a learnable nonlinear function.

Stacking the logits across all interventions produces a three-dimensional tensor ${\bf{L}}\in {{\mathbb{R}}}^{B\times 4\times K},$ where the second dimension indexes the four categorical states and the third dimension indexes the interventions. Each function ${f}_{k}$ is implemented as a shallow feed-forward network with one hidden layer and nonlinear activation, allowing flexible nonlinear mappings from the shared intervention embedding to the four state scores.

#### Temporal decoder head

For each intervention k, the model estimates the probability of administration over the 6-hour horizon:

14 $${{\bf{p}}}_{k}=\sigma ({g}_{k}({{\bf{F}}}_{{\rm{out}}}[:,k,:]))\in {[0,1]}^{B\times 6}$$

where ${g}_{k}:{{\mathbb{R}}}^{H}\to {{\mathbb{R}}}^{6}$ is a learnable nonlinear function and $\sigma (\cdot )$ denotes the element-wise sigmoid.

Stacking across interventions produces ${\bf{P}}\in {[\mathrm{0,1}]}^{B\times 6\times K}$, where ${p}_{t,k}$ represents the probability that the intervention *k* is administered at prediction hour t. Each ${g}_{k}$ is implemented as a shallow feed-forward network analogous to the discrete head, but mapping to a six-dimensional output corresponding to the prediction horizon.

Because the two decoder variants address different prediction tasks and class-imbalance profiles, separate loss functions were used for optimisation.

#### Discrete decoder (weighted cross-entropy loss)

For categorical state prediction, we employ *weighted cross-entropy* to address class imbalance. Class weights are inversely proportional to the prevalence of each class:

15 $${w}_{c}=\frac{N}{C\cdot {n}_{c}}$$

where *N* is the total number of samples, C=4 is the number of classes (ONSET, WEAN, STAY-ON, STAY-OFF), and *n*_*c*_ is the number of samples in class c. This weighting ensures that minority classes contribute proportionally to the loss despite their lower prevalence. The discrete decoder loss for a single sample is then computed as:

16 $${{\mathcal{L}}}_{\mathrm{CE}}=-\mathop{\sum }\limits_{c=1}^{C}{w}_{c}\,{y}_{c}\log ({\hat{y}}_{c})$$

where *y*_*c*_ and ${\hat{y}}_{c}$ are the true and predicted probabilities for class c, respectively.

#### Temporal decoder (Focal Loss)

For hourly intervention probability prediction, we employ focal loss^79^, a method developed by Facebook AI Research to handle extreme class imbalance in object detection tasks. Focal loss down-weights well-classified examples and focuses learning on hard, minority-class cases. For our prediction of intervention probabilities ${{\bf{p}}}_{t,k}$ at hour *t*for intervention k, the focal loss is defined as:

17 $${{\mathcal{L}}}_{\mathrm{focal}}=-\mathop{\sum }\limits_{t=1}^{T}\mathop{\sum }\limits_{k=1}^{K}[\alpha (1-{p}_{t,k}{)}^{\gamma }{y}_{t,k}\log ({p}_{t,k})+(1-\alpha ){({p}_{t,k})}^{\gamma }(1-{y}_{t,k})\log (1-{p}_{t,k})]$$

where T=6 is the number of prediction hours, K=7 is the number of interventions, ${y}_{t,k}\in \{\mathrm{0,1}\}$ is the ground truth label, γ=2 is the focusing parameter, and α=0.25 balances positive and negative classes. This formulation allows the model to focus on rare intervention events while down-weighting the contribution of well-predicted “no intervention” cases, which dominate the ICU dataset.

To enable interpretable, patient-level risk stratification within the FIRST-ICU framework, we incorporated a probabilistic post-prediction layer based on Generative Topographic Mapping (GTM)^66^, a principled non-linear dimensionality reduction method that projects high-dimensional predicted intervention trajectories into a smooth, low-dimensional latent space while preserving topological relationships between data points. GTM addresses key limitations of deterministic visualisation methods by providing a complete probabilistic framework with explicit likelihood-based training via the expectation-maximisation algorithm^66^, enabling both uncertainty quantification and direct out-of-sample projection of new patient data onto the trained manifold. These capabilities are critical for real-time clinical deployment. In addition, GTM has been successfully applied to patient phenotyping in prior studies^80,81^, demonstrating its ability to uncover clinically meaningful clusters from high-dimensional clinical data. Unlike non-parametric visualisation methods such as t-SNE or UMAP, GTM provides a probabilistic mapping, supports uncertainty estimation, and allows the projection of unseen patient data onto the trained latent space, making it well-suited for prospective clinical deployment. Comparative studies have demonstrated that GTM outperforms alternative dimensionality reduction techniques in preserving both local and global structure while maintaining interpretability in clinical applications^82–84^.

Formally, let the outputs of the temporal decoder for a batch of patients be represented as a three-dimensional tensor:

18 $$P\in {{\mathbb{R}}}^{B\times T\times K}$$

where *B*is the batch size, *T* is the prediction horizon in hours, and *K* is the number of interventions. Each patient’s temporal predictions ${P}_{b}$ are flattened into a single vector:

19 $${x}_{b}={\rm{vec}}({P}_{b})\in {{\mathbb{R}}}^{T\cdot K},b=1,\ldots ,B$$

and log-transformed for normalisation:

20 $${\widetilde{x}}_{b}=\log ({x}_{b}+\epsilon )$$

to mitigate skewness and stabilise scale.

GTM assumes each ${\widetilde{x}}_{b}\in {{\mathbb{R}}}^{D}$(here D=T⋅K) is generated from a low-dimensional latent variable $u\in {{\mathbb{R}}}^{2}$ via a smooth nonlinear mapping:

21 $$y({u;W})=W\,\phi (u)$$

where $\phi (u)\in {{\mathbb{R}}}^{M}$ is a vector of radial basis functions (RBFs) evaluated at u, and $W\in {{\mathbb{R}}}^{D\times M}$ is a weight matrix learned from the data. The data density is modelled as a constrained mixture of Gaussians centred at the mapped latent nodes:

22 $$p({\widetilde{x}}_{b}| W,\beta )=\frac{1}{L}\mathop{\sum }\limits_{i=1}^{L}{\mathscr{N}}{\mathscr{(}}{\widetilde{x}}_{b}| y({u}_{i}{;W}),{\beta }^{-1}I)$$

where $L={k}^{2}$ is the number of latent nodes arranged on a *k* × *k*grid, and *β* is the Gaussian precision. Optimisation maximises the log-likelihood of the data under this mixture, constrained by a regularisation factor *λ* to prevent overfitting. Each latent node defines a reference vector in data space, forming a probabilistic membership map:

23 $$p(l| {\widetilde{x}}_{b})=\frac{{\mathcal{N}}\left({\widetilde{x}}_{b},|,{y}_{l},{\beta }^{-1}I\right)}{{\sum }_{j=1}^{L}{\mathcal{N}}{\mathscr{(}}{\widetilde{x}}_{b}| {y}_{j},{\beta }^{-1}I)}$$

allowing probabilistic assignment of each patient to regions of the latent space while preserving neighbourhood structure. Hyperparameters tuned in this framework include the latent grid size k, the number of RBFs M, the RBF width s, and the regularisation factor λ. These were selected via grid search to maximise data log-likelihood on the training set while maintaining interpretable cluster structure, as described in the GTM-based risk stratification experiments.

To facilitate clinical interpretation, the fine-grained GTM reference vectors $\left\{{y}_{1},\ldots ,{y}_{L}\right\}$ were subsequently grouped into macro-clusters representing coherent intervention phenotypes, such as escalating vasopressor support, ventilatory failure, or stable trajectories. Agglomerative hierarchical clustering with Ward’s minimum variance criterion^80,81,85^ provides a principled approach to form such macro-clusters, minimising within-cluster variance:

24 $${C}_{m}={\mathrm{arg}}\,{\mathrm{min}}\mathop{\sum }\limits_{i\in {C}_{m}}\parallel {y}_{i}-{\mu }_{m}{\parallel }^{2},{\mu }_{m}=\frac{1}{{\rm{| }}{C}_{m}{\rm{| }}}\mathop{\sum }\limits_{i\in {C}_{m}}{y}_{i}$$

where ${\mu }_{m}$ is the cluster centroid. Patient outcomes were then overlaid post hoc on these macro-clusters to identify high-risk regions without influencing GTM training, enabling population-level risk stratification directly from predicted intervention patterns. Each patient was probabilistically assigned to macro-clusters via the GTM posterior, allowing individual predicted trajectories to be mapped to population-level phenotypes and high-risk regions.

### Experiments

All deep learning models were trained using a common optimisation procedure based on mini-batch gradient descent with the Adam optimiser (initial learning rate ${10}^{-4}$). Early stopping based on validation loss, with a patience of 5 epochs, was used to prevent overfitting, and learning rate decay (factor 0.5) was applied when validation loss plateaued. The batch size was set to 64 for all models. The dataset was split into 80% for training and 20% for testing, with a validation subset randomly sampled from 20% of the training set for hyperparameter tuning. Identical train/test splits were used across baseline and proposed models to ensure fair comparison. Evaluation metrics were then selected according to the output structure of each decoder variant.

#### Discrete decoder metrics

For categorical state prediction, we report Macro AUC-PR, Macro F1, and Macro Average Precision, computed per intervention and averaged across all seven interventions. To ensure fair and interpretable comparison across interventions with varying prevalence, we optimised a decision threshold for each intervention independently by selecting the threshold that maximised the F1 score on the validation set. Predicted class labels were obtained by dividing the predicted probabilities by the respective optimal thresholds and selecting the class with the maximum adjusted score. This normalised thresholding procedure ensures that F1 and related metrics reflect balanced performance across interventions, particularly for minority classes such as ONSET and WEAN events.

Confidence intervals for all macro metrics were estimated using 1000 bootstrap resamples, providing uncertainty quantification for model performance. Metrics were chosen to emphasise precision-recall trade-offs as ICU intervention classes are highly imbalanced and precision-recall metrics are more sensitive to performance on the clinically important minority classes.

#### Temporal decoder metrics

For hourly probability prediction, we report: AUC-ROC (Area Under the Receiver Operating Characteristic Curve), measuring discrimination ability across all classification thresholds; AUC-PR, particularly important given class imbalance; Brier Score, quantifying both calibration and discrimination as the mean squared error between predicted probabilities ${p}_{i}$ and binary outcomes ${y}_{i}$:

25 $${\rm{BS}}=\frac{1}{N}\mathop{\sum }\limits_{i=1}^{N}{({p}_{i}-{y}_{i})}^{2}$$

Finally, we report reliability calibration curves, providing a visual assessment of calibration by plotting observed event frequencies against predicted probabilities, with quantification via the expected calibration error (ECE).

Confidence intervals for all metrics were calculated using bootstrapping with 1000 iterations^86^, providing uncertainty estimates for performance comparisons.

To contextualise the performance of FIRST-ICU, we compared it against established state-of-the-art baselines from the literature. All baseline methods were evaluated only against the Discrete Decoder to ensure a like-for-like comparison on categorical intervention state prediction. Importantly, while all baselines adopt single-task learning by training a separate model for each intervention, FIRST-ICU performs joint multi-task prediction across all seven interventions, enabling explicit modelling of co-administration and treatment interactions. To ensure fairness at the optimisation level, all baseline models were retrained using the same weighted cross-entropy loss employed by the discrete decoder, with class weights computed from the training data. This ensures that performance differences arise from modelling capacity rather than from differences in loss formulation or class-imbalance handling. Parameter counts for all models were retrieved to contextualise performance differences relative to model complexity.

The approach from Suresh et al.^29^ (LSTM and CNN**)** trains independent models per intervention, using either recurrent or convolutional architectures. The LSTM variant consists of two hidden layers with 512 units each and processes 6-h input windows to predict one of four categorical intervention states after a 6-hour gap. The original model incorporates heterogeneous inputs, including 29 time-varying vital signs and laboratory measurements, 5 static patient variables, and 50-dimensional topic vectors derived from clinical notes using Latent Dirichlet Allocation (LDA). To ensure consistency with the structured-data focus of FIRST-ICU and to avoid confounding from free-text information, we trained this baseline using only the structured clinical variables, excluding the note-derived topic vectors. The CNN variant applies temporal convolutions at three granularities (kernel sizes of 3, 4 and 5 h), each with 64 filters, followed by max-pooling and two fully connected layers.

This model from Xu et al.^22^ (GCNN) integrates temporal convolution (TC) and graph convolution (GC) in an alternating architecture. The TC blocks employ dilated causal convolutions with multiple kernel sizes arranged in an inception-style module, enabling the extraction of multi-scale temporal patterns. The dilation factor increases exponentially across layers $(d={2}^{n})$, yielding a receptive field of approximately 125 h after six layers. The GC component uses mix-hop propagation layers and an adaptive graph learning module that learns the inter-variable adjacency matrix during training, allowing dynamic modelling of physiological relationships.

To assess the generalisability of FIRST-ICU temporal trajectory prediction across institutions and healthcare systems, we performed external validation using the AmsterdamUMCdb^10^. Because some variables available in MIMIC-IV were not recorded in AmsterdamUMCdb, including ethnicity (5 indicators), capillary refill time, patient weight, and prothrombin time, the MIMIC-IV cohort was re-extracted using only the subset of features shared by both datasets. This ensured identical input dimensionality and prevented spurious performance differences arising from feature availability rather than model generalisation. This harmonisation strategy also demonstrates the adaptability of FIRST-ICU to heterogeneous clinical databases, which is essential for real-world deployment across hospitals with differing documentation practices^24,87^.

Given the differences in treatment protocols between North America and Europe^42,43^, external validation was formulated in terms of physiological treatment needs rather than specific drug choices. Specifically, invasive and non-invasive ventilation were merged into a single respiratory failure (ventilation need) target, vasopressor agents including phenylephrine, epinephrine, norepinephrine, and dopamine were aggregated into a distributive or hypovolemic shock (vasopressor need) category, and dobutamine was used to represent cardiogenic shock (inotrope need)^37,39,42^. This abstraction enables cross-continental validation of clinically meaningful states even when local prescribing practices differ, allowing the model to be evaluated on whether it correctly predicts the need for respiratory, circulatory, or cardiac support rather than the exact pharmacological agent administered.

The Temporal Decoder trained on MIMIC-IV was applied directly to the AmsterdamUMCdb test set without retraining, recalibration, or fine-tuning, providing a stringent assessment of out-of-distribution generalisation. Performance was evaluated using AUC-ROC and AUC-PR to quantify discrimination under severe class imbalance, and the Brier score to assess probabilistic calibration and overall predictive accuracy. Together, these metrics quantify both the ability of FIRST-ICU to rank patients by risk and the reliability of its predicted intervention probabilities across healthcare systems.

To quantify the contribution of the IIAM, we conducted an ablation study on the Temporal Decoder. In the ablated variant, the LSTM outputs are passed directly to the prediction heads, bypassing the IIAM. This removes the temporal attention mechanism used to weight informative timesteps, the shared normalised feature encoder, and the multi-head self-attention layer that models dependencies between interventions.

This comparison isolates the performance gain attributable to the IIAM and validates our architectural design decisions. The ablation study uses identical training procedures, hyperparameters, and evaluation metrics to ensure a fair comparison. By comparing the full model against the ablated variant across all interventions and prediction timepoints using AUC-PR as the primary metric, we quantify the specific contribution of: (1) temporal attention for identifying the most relevant observation periods; (2) normalised feature transformation for stable training; and (3) explicit modelling of intervention co-occurrence patterns through self-attention.

To rigorously evaluate the model’s ability to predict intervention onset from physiological signals alone, without relying on intervention history, we conducted a focused analysis on cold-start prediction cases. These are defined as context windows where: (1) No instance of intervention k was administered during the observation window, (2) At least one instance of intervention k was administered during the prediction window.

This subset represents the clinically most challenging and valuable prediction scenario: anticipating intervention needs before any treatment has been initiated. By isolating these cases, we assess whether the model learns genuine physiological precursors to intervention (e.g. declining SpO_2_ before ventilation, rising lactate before vasopressors) rather than simply extrapolating current intervention states.

This analysis was performed on both the MIMIC-IV test set and the AmsterdamUMCdb external validation cohort. Because the objective in this setting is to identify true impending intervention events rather than to rank all time windows by relative risk, performance was assessed primarily using AUC-PR and the Brier score. Given the lower prevalence of onset events compared to the full test set, confidence intervals were computed using bootstrapping with 1000 iterations in both cohorts.

To assess the interpretability and clinical utility of the predicted intervention trajectories, we conducted experiments using the GTM-based post-prediction layer for patient-level and population-level risk stratification. The goal was to determine whether GTM captures coherent intervention patterns and identifies high-risk groups without using outcome information during training. Flattened, log-transformed predicted intervention probabilities (T⋅K dimensional vectors representing T-hour trajectories across K interventions) from the Temporal Decoder were embedded into a two-dimensional latent space using GTM. We trained GTM on the training cohort using the following hyperparameters: latent grid size *k* (yielding L=k*k latent nodes), *M* RBFs, RBF width s, and regularisation factor λ. These parameters were selected via grid search to maximise data log-likelihood while maintaining visually interpretable cluster structure on validation data.

For clinical interpretation, GTM reference vectors were grouped into macro-clusters using agglomerative hierarchical clustering with Ward’s minimum variance criterion. We evaluated cluster solutions ranging from 3 to 10 macro-clusters and selected the optimal number based on clinical interpretability assessed by two independent critical care physicians.

Clinical relevance was then assessed by overlaying mortality outcomes on the macro-clusters post hoc. Importantly, mortality was not used during GTM fitting or clustering, ensuring that high-risk regions reflect associations emergent from predicted intervention trajectories rather than outcome-driven clustering. For each macro-cluster, we calculated the mortality-associated trajectory percentage by dividing the number of trajectories linked to patients who died by the total number of trajectories in that cluster. Clusters with elevated mortality percentages relative to the overall cohort baseline were designated as high-risk phenotypes.

At the patient level, we examined representative trajectories from each macro-cluster to compare predicted intervention trajectories with the observed clinical course. For each sampled patient, we compared their predicted intervention trajectories against the actual interventions administered during the corresponding time window. The predicted trajectories consisted of 6-hour intervention probability sequences generated by the Temporal Decoder, while observed patterns were extracted from the clinical record during the same temporal window.

We visualised individual patient positions on the GTM two-dimensional latent space map, with colour-coding indicating macro-cluster assignment and mortality outcome. This visualisation allows for spatial interpretation of where each patient’s predicted trajectory falls relative to established intervention phenotypes and high-risk regions. Patients positioned near cluster boundaries represent cases with mixed or transitional intervention patterns, while those in cluster cores exhibit prototypical phenotype characteristics.

To quantify alignment between predictions and observations, we assessed clinical concordance by examining agreement between predicted escalation or de-escalation patterns and the observed clinical course. This analysis considers both the direction of intervention changes (escalation vs. de-escalation vs. stability) and the specific intervention types involved. By mapping individual patient trajectories to their positions on the membership map, this interpretability layer within FIRST-ICU bridges predictive performance with actionable understanding, supporting both personalised trajectory analysis and population-level decision support.

#### Ethics statement

The present study was conducted in accordance with the Declaration of Helsinki and was based exclusively on de-identified or pseudonymised data obtained from publicly available databases (MIMIC-IV and AmsterdamUMCdb). As the present work involved secondary analysis of existing de-identified data, no additional informed consent was required.

## Supplementary information

Supplementary information

## Data Availability

MIMIC-IV is available via PhysioNet (https://physionet.org/content/mimiciv/). AmsterdamUMCdb is available via Amsterdam Medical Data Science (https://amsterdammedicaldatascience.nl/). Both require credentialing and data use agreements.

## Code availability

The code for FIRST-ICU is available on https://doi.org/10.5281/zenodo.19609882
