# Supplementary material for: FIRST-ICU: forecasting interventions and risk stratification in the ICU using graph neural network autoencoders
Source: NPJ Digit Med. 2026 Jun 11;9:722. doi: 10.1038/s41746-026-02890-1 (PMC13601581; doi:10.1038/s41746-026-02890-1)

SUPPLEMENTARY MATERIAL

Supplementary Figure 1. Calibration of intervention probability forecasts. Reliability diagrams for the seven ICU interventions, aggregated across prediction horizons from 1 to 6 hours ahead (T=19-24). Each panel shows the relationship between the mean predicted probability (x-axis) and the observed event frequency (y-axis), with the dashed diagonal indicating perfect calibration. Curves closer to the diagonal indicate better agreement between predicted risks and empirical outcomes. The Brier score (BS) reported in each panel summarises overall probabilistic accuracy, with lower values indicating better calibrated and more accurate predictions. All interventions demonstrate good calibration with BS < 0.035.

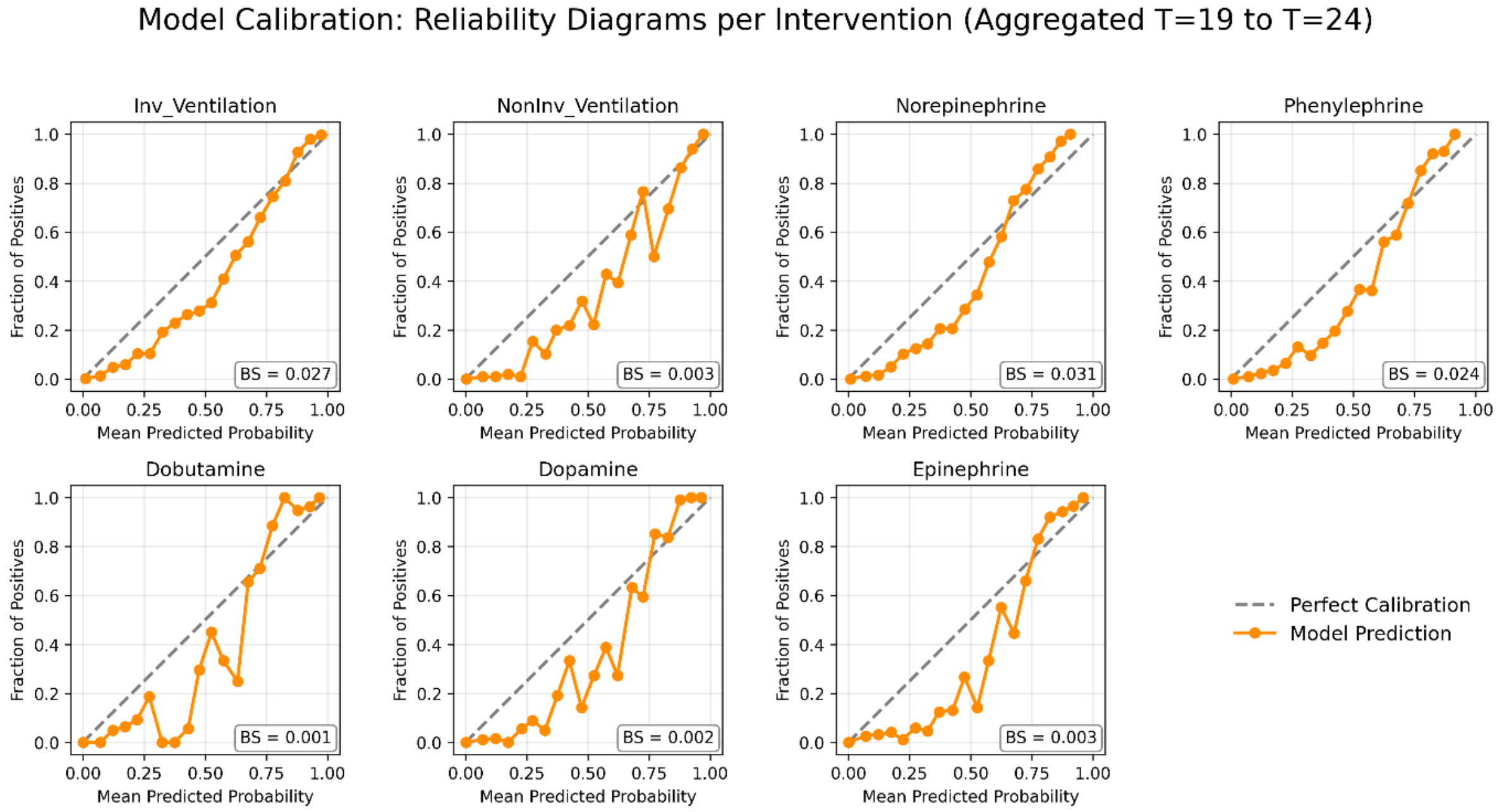

Supplementary Figure 2. Cluster number selection: silhouette scores across K=2 to K=8 macro-clusters. Silhouette scores computed on GTM reference vectors clustered via agglomerative hierarchical clustering with Ward's criterion and k-nearest-neighbour connectivity constraint. Scores plateau across K=4 to K=8 (range 0.306-0.310), with no single solution statistically dominant. The K=6 solution (silhouette score = 0.308, red marker) was selected based on independent clinical review by two critical care physicians. Stability of the K=6 solution was confirmed across five random GTM initialisations (Adjusted Rand Index = 1.000 for all pairwise comparisons).

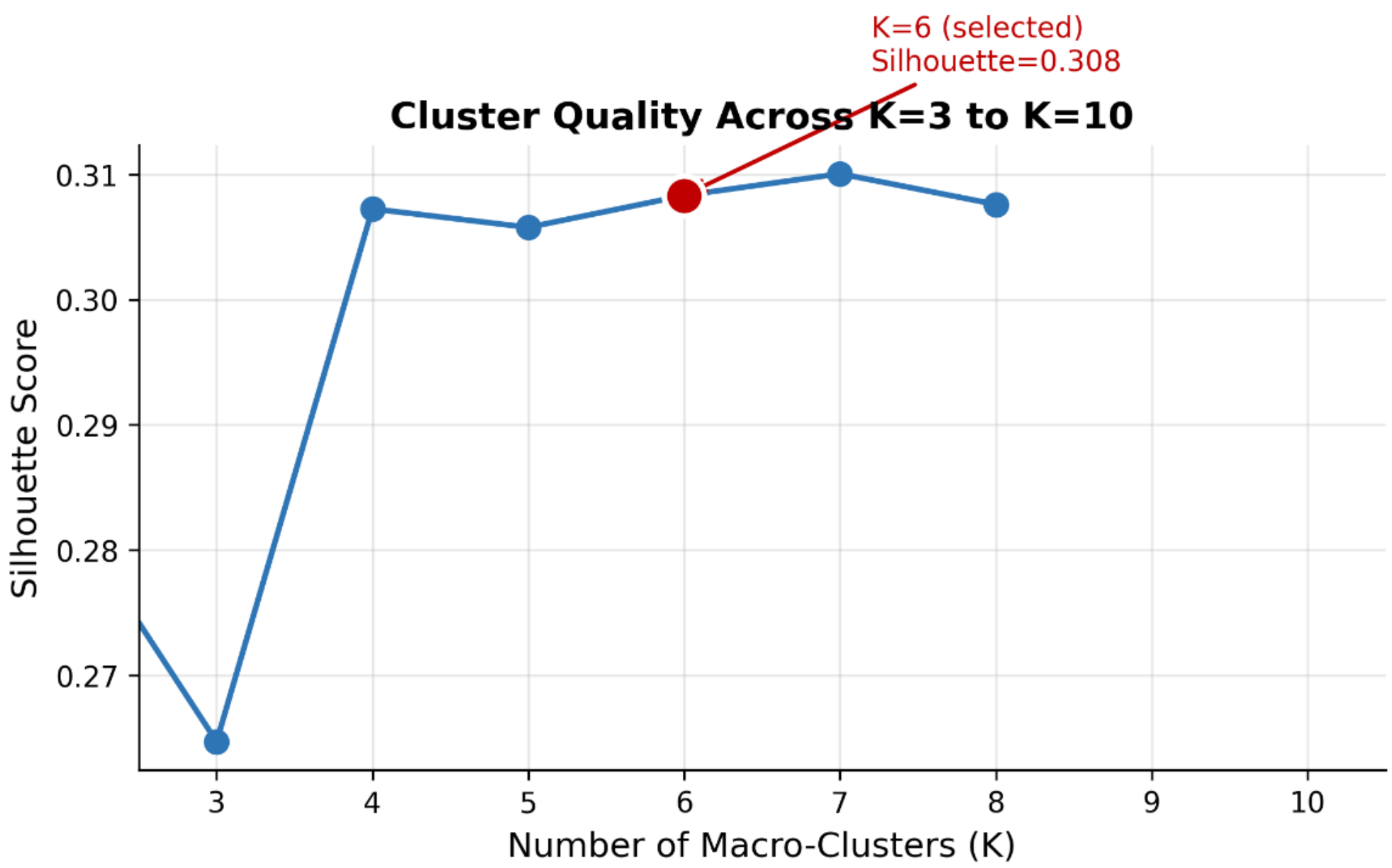

Supplement: Supplementary file 1 [file 41746_2026_2890_MOESM1_ESM.pdf]
